# Systematic analysis of human telomeric dysfunction using inducible telosome/shelterin CRISPR/Cas9 knockout cells

**DOI:** 10.1038/celldisc.2017.34

**Published:** 2017-09-26

**Authors:** Hyeung Kim, Feng Li, Quanyuan He, Tingting Deng, Jun Xu, Feng Jin, Cristian Coarfa, Nagireddy Putluri, Dan Liu, Zhou Songyang

**Affiliations:** 1 Verna and Marrs McLean Department of Biochemistry and Molecular Biology, Baylor College of Medicine, Houston, TX, USA; 2 Key Laboratory of Gene Engineering of the Ministry of Education and State Key Laboratory for Biocontrol, School of Life Sciences, Sun Yat-sen University, Guangzhou, China; 3 Cell-Based Assay Screening Service Core, Baylor College of Medicine, Houston, TX, USA; 4 Department of Molecular and Cellular Biology and Advanced Technology Core, Baylor College of Medicine, Houston, TX, USA

**Keywords:** CRISPR/Cas9, inducible knockout, metabolism, POT1 isoform, telomere, telosome/shelterin

## Abstract

CRISPR/Cas9 technology enables efficient loss-of-function analysis of human genes using somatic cells. Studies of essential genes, however, require conditional knockout (KO) cells. Here, we describe the generation of inducible CRISPR KO human cell lines for the subunits of the telosome/shelterin complex, TRF1, TRF2, RAP1, TIN2, TPP1 and POT1, which directly interact with telomeres or can bind to telomeres through association with other subunits. Homozygous inactivation of several subunits is lethal in mice, and most loss-of-function studies of human telomere regulators have relied on RNA interference-mediated gene knockdown, which suffers its own limitations. Our inducible CRISPR approach has allowed us to more expediently obtain large numbers of KO cells in which essential telomere regulators have been inactivated for biochemical and molecular studies. Our systematic analysis revealed functional differences between human and mouse telomeric proteins in DNA damage responses, telomere length and metabolic control, providing new insights into how human telomeres are maintained.

## Introduction

In the past 20 years, we have gained tremendous insight into how the ends of mammalian chromosomes or telomeres are maintained and regulated. Together with the telomerase, which consists of the reverse transcriptase TERT and RNA template TR/TERC, a multitude of telomere-binding proteins participate in telomere maintenance [1–5]. In particular, six core telomeric proteins, TRF1, TRF2, RAP1, TPP1, TIN2 and POT1, dynamically assemble on telomeres as a large complex called telosome or shelterin and are essential in telomere length regulation and end protection in mammals [6–8]. Extensive research has revealed the interactions and functions of telosome components. For instance, TRF1 and TRF2 bind directly to the telomere duplex through their myb domains [9–13], whereas POT1 binds 3’ single-stranded (ss) telomeric overhangs [14, 15]. RAP1 is recruited by TRF2, but apparently does not directly interact with any of the other subunits [16]. TIN2 can interact with both TRF1 and TRF2 [6, 17–21]. It also binds TPP1 and helps bring to telomeres the TPP1-POT1 heterodimer that is essential for regulating telomerase access to telomeres [21–30]. The core telomere proteins often act as interaction hubs to recruit factors of diverse pathways to telomeres and ensure crosstalk between telomere maintenance pathways and other cellular processes [8, 19, 31, 32]. In fact, several key telomere regulators have been shown to regulate metabolism, providing direct evidence of the close ties between telomere regulation and metabolic control. For example, the human telomerase reverse transcriptase has been found to localize to the mitochondria and reduce intracellular oxidative stress [33–36]. Our lab has found that TIN2 can also localize to the mitochondria and regulate oxidative phosphorylation [37].

Numerous studies have demonstrated that dysfunctional telomeres can lead to telomere length defects, deprotected telomeres, genomic instability and diseases [1, 4, 32, 38]. Much of our knowledge regarding the molecular and functional significance of mammalian telomeric proteins comes from studies using mouse knockout (KO) mouse embryonic fibroblast (MEF) cells, as genes are more readily targeted in mouse embryonic stem cells. However, notable differences exist in telomere regulation between mouse and human. For instance, human telomeres are considerably shorter than those of laboratory mice and human has one *POT1* gene, whereas mouse has two (*Pot1a* and *Pot1b*). Such disparities underscore the need for loss-of-function human cellular models. Majority of the loss-of-function studies in human cells have relied on RNA interference (RNAi)-mediated inhibition of endogenous genes. The limitations of RNAi knockdown (KD) and the fact that several key telomere regulators including TRF2 and TIN2 are essential genes have complicated data analysis and interpretation. Complete inactivation of these telomere regulatory genes in cells may cause cell death, precluding further detailed biochemical and molecular studies, especially experiments that require extended culturing and/or large numbers of cells.

The advent of the CRISPR/Cas9 genome-editing technology has afforded investigators unprecedented opportunities to more efficiently and specifically target genes in human cells and to explore the consequences of their inactivation [39–47]. In this study, we took advantage of the highly flexible and adaptable CRISPR/Cas9 system and generated human inducible KO cell lines for each of the telosome components. This panel of cells has allowed us to survey the functional significance of each telomeric protein and probe the impact of individual subunit inhibition on telomere regulation as well as metabolic control. With this systematic analysis of the function of human telomere proteins using inducible KO cell lines, we are able to better delineate the differences between mouse and human telomere biology. In addition, our panel of inducible KO cell lines should prove invaluable to investigators seeking to further explore the consequences of telomere dysfunction and to study how diverse cellular functions may be disrupted upon telomere dysregulation.

## Results

### Using CRISPR/Cas9 to generate inducible KO human cell lines

*Trf1*, *Trf2* and *Tin2* have been reported to be essential genes in mouse [48–50]. To determine the roles of their human orthologs, we first turned to RNAi KD in human cells through stable expression of short hairpin RNAs (Supplementary Figure S1A). Even with effective KD (>80%) of TRF2, for example, we could only observe minor DNA damage responses (DDRs) at telomeres (data not shown), rarely more severe phenotypes such as chromosome end-to-end fusions found in *Trf2* KO MEF cells [50], suggesting that residual TRF2 proteins in the KD cells may have been sufficient to prevent severe and sustained telomere DNA damages. We next attempted straight KO of these genes by CRISPR/Cas9, but failed to isolate any clones of TRF2, TIN2 or POT1 KO cells. Given such findings, we decided that human cells conditionally knocked out for telosome subunits would be more desirable.

Traditionally, conditional KO alleles are generated by inserting into some particular locus recombination sequences such as *loxp* sites, which can mediate the deletion of intervening sequences upon the expression of recombinases such as Cre [51–53]. The insertion of such exogenous sequences may alter gene regulation and the entire process is often time consuming. To generate conditional telosome subunit KO cells, we modified a lentivirus-based inducible CRISPR/Cas9 KO system [54], and constructed separate vectors for inducible Cas9 and constitutive single guide RNA (sgRNA) expression (Supplementary Figure S1B). Hela cells were transduced with lentiviruses encoding inducible Cas9 and a clone in which Cas9 expression could be reproducibly activated with doxycycline in a dose-dependent manner was selected (Supplementary Figure S1C).

**Figure 1:**
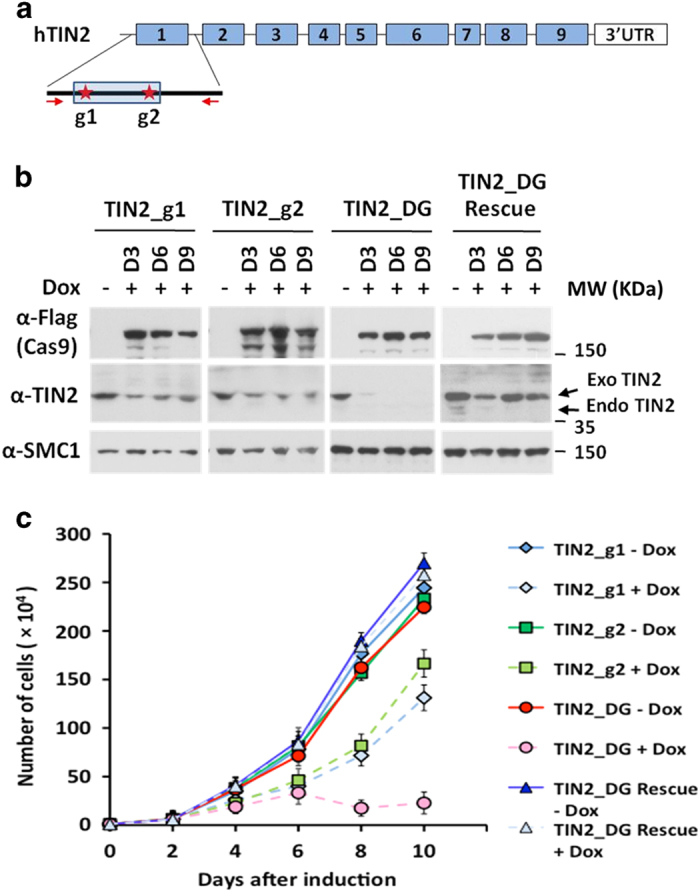
The dual sgRNA strategy enables more complete inactivation of endogenous genes. (**a**) Schematic representation of the TIN2 locus. Shaded boxes indicate exons and sites targeted by the sgRNAs (g1 and g2) are marked. The distance between the two predicted cleavage sites (red asterisks) is 147 bps. Red arrows indicate positions of PCR primers for genomic sequence verifications. (**b**) Cas9-inducible Hela cells stably expressing single (TIN2_g1 or TIN2_g2) or dual sgRNAs (TIN2_DG) were induced with 1 μgml^−1^ doxycycline for 3, 6 or 9 days, and then collected for immunoblotting using the indicated antibodies. TIN2_DG cells that also stably expressed sgRNA-resistant Flag-tagged TIN2 (Rescue) were also included. The anti-SMC1 antibody served as a loading control. The expected sizes for endogenous (Endo) and exogenous (Exo) TIN2 are indicated by arrows. (**c**) Cells from **b** were collected at the indicated time points following addition of doxycycline (Dox). The number of live cells was determined by Trypan blue exclusion. At least three experiments with independent doxycycline inductions were performed and the results were combined and plotted as indicated. Error bars indicate s.e.

The double-strand breaks resulting from Cas9 cleavage trigger the non-homologous end joining DNA repair pathway in the absence of a donor template [55–57]. Non-homologous end joining-mediated DNA repair may generate small insertions and/or deletions (indels) at the target site, and compromise gene function if cleavage occurs within protein coding sequences. Repair of a single Cas9 cleavage site has a 1/3 chance of in-frame ligation of the coding sequences, which may not completely disrupt gene function. We reasoned that simultaneous targeting with two sgRNAs should improve the odds of larger deletions and more complete inhibition of endogenous genes. To test this strategy, the inducible Cas9 cells were infected with two viruses encoding two separate TIN2-specific sgRNAs either singly or together, selected with appropriate antibiotics, and then cultured in doxycycline-containing media to induce Cas9 expression (Figure 1a). At different time points following doxycycline treatment, cells were collected for analysis of TIN2 protein expression (Figure 1b). As we predicted, targeting with two sgRNAs appeared to KO gene expression more efficiently than using a single sgRNA. Furthermore, lengthier doxycycline treatment was able to improve KO efficiency (Figure 1b).

Notably, the TIN2 KO cells exhibited proliferative defects during culturing (Figure 1c). Although all of the cell lines showed similar growth patterns in the absence of doxycycline, differences in growth rates became apparent between doxycycline-induced TIN2 KO cells after 4-day treatment. Growth of the single sgRNA TIN2 KO cells was hampered initially, but appeared to recover with continued culturing, likely due to the presence of cells with incomplete TIN2 inhibition. Indeed, the severity of proliferation defects correlated with the degree of TIN2 ablation, with the dual sgRNA TIN2 KO cells being more severely affected than the single sgRNA KO cells (Figure 1c). When we ectopically expressed sgRNA-resistant TIN2 in the dual sgRNA KO cells, growth and proliferation were restored, indicating that TIN2 was critical for cell growth and that dual sgRNAs more completely knocked out TIN2.

Although the inducible TIN2 KO cells were polyclonal, independent inductions of Cas9 led to highly reproducible results, indicating that the inducible strategy reliably produces populations of cells with comparable genotypes and phenotypes. When we sampled the TIN2 alleles from the induced dual sgRNA TIN2 KO cells by TOPO cloning and Sanger sequencing of the sgRNA target region (Supplementary Figure S2A), we found most (>80%) to contain deletions because of simultaneous Cas9 cleavage at both sgRNA target sites, and the remaining alleles containing indels at both target sites without deleting the intervening sequences. Importantly, all of the alleles are predicted to have impaired TIN2 function, corroborating that dual sgRNA design helped ensure complete inactivation of endogenous genes.

Using the dual sgRNA system, we generated inducible KO cell lines for all six core telomeric proteins (Supplementary Figure S2B). Again, we compared single vs dual sgRNA KO efficiencies. Although some of the single sgRNAs knocked out endogenous gene expression quite effectively, using dual sgRNAs to simultaneously target a single locus consistently proved more efficient (Supplementary Figure S2C). Again, longer doxycycline induction led to more effective and sustained inactivation of endogenous genes (Supplementary Figure S2C). In the following experiments, all the cell lines were induced for 6 days with doxycycline before further analysis and/or treatment unless otherwise specified.

### Profiling the contribution of each subunit to the telomeric assembly of the telosome complex

**Figure 2:**
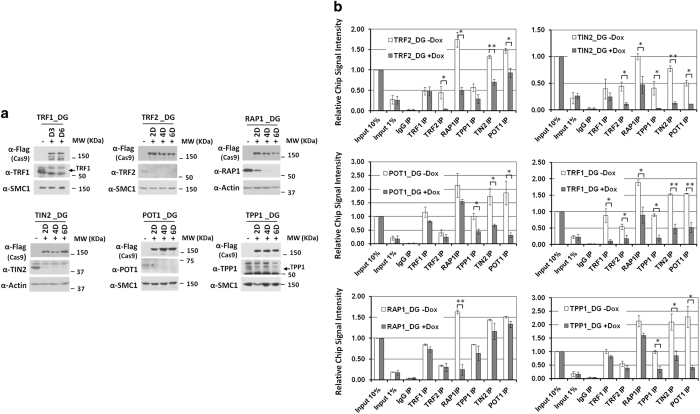
Removal of individual subunits impacts the organization of the telosome. (**a**) For each subunit of the telosome, we generated inducible Cas9 cells stably expressing two sgRNAs targeting the gene. Cells were treated with doxycycline for up to 6 days before being analyzed by western blotting using the indicated antibodies. Anti-SMC1 and -actin antibodies served as loading controls. Molecular weight makers (kDa) are indicated on the right. (**b**) Cells from **a** (after 6 days of induction) were immunoprecipitated using appropriate antibodies to bring down associated telomeric DNA for dot blotting and hybridization with a telomere repeat probe (TTAGGG)_3_. Signals for each cell line were quantified and normalized against input. At least three experiments with independent doxycycline inductions were performed for each cell line, and the results were combined and plotted as indicated. Error bars indicate s.e. *P-*values were obtained using the Student’s *t*-test. *P<0.05, **P<0.01.

The six KO cell lines afforded us the first opportunity to systematically investigate in detail how constitutive deletion of one subunit may affect the telomeric targeting and assembly of the telosome complex. Each cell line was induced and confirmed for KO efficiency by western blotting (Figure 2a). The cells were then harvested for telomere chromatin immunoprecipitation (ChIP) assays (Supplementary Figure S3A).

Of the six proteins, both TRF1 and TRF2 can bind double-stranded telomeric DNA [9–13], and as expected, we found that TRF2 KO had no effect on TRF1 binding to telomeres (Figure 2b). Using ectopically expressed proteins, we showed previously that TIN2 was essential for telosome assembly [23]. Although POT1 can bind ss telomeric DNA [14, 15], targeting of POT1 to telomeres requires TPP1, which in turn is tethered to telomeres through TIN2 [23, 24]. Consistent with these previous findings, reductions in telomere targeting of the remaining subunits were apparent in TIN2 KO cells, with TPP1 and POT1 being the most affected (Figure 2b). Similarly, knocking out TPP1 also led to drastic reductions in telomere ChIP signals for other telosome subunits, particularly POT1, underscoring the importance of TPP1 in telosome assembly and POT1 telomere targeting [24, 25]. Notably, POT1 KO also significantly reduced the targeting of both TPP1 and TIN2 to telomeres. Taken together, these data suggest that TIN2, TPP1 and POT1 may form a tight subcomplex. It is also clear that with the exception of RAP1, knocking out any of the subunits had a more global effect on the remaining subunits (Figure 2b), indicating significant contribution of each protein to the proper assembly of the telosome complex and that their roles in maintaining telosome function may be more complex than previously surmised.

### Activation of telomere DDRs in inducible KO cell lines

**Figure 3:**
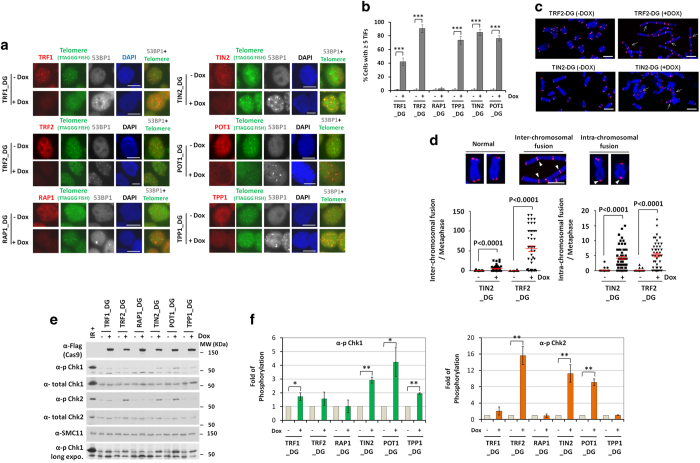
Deletion of individual subunits impacts telomere end protection. The inducible KO cell lines were maintained in the presence (+) or absence (−) of doxycycline for 6 days before being collected and used in the following assays. (**a**) The cells were examined by IF-fluorescence *in situ* hybridization (FISH) using antibodies against 53BP1 and the respective targeted proteins along with a telomere PNA probe. 4,6-Diamidino-2-phenylindole (DAPI) was used to stain the nuclei. Scale bars 10 μm. (**b**) Data from **a** were quantified and graphed. Three experiments with independent doxycylcine inductions were carried out for each cell line, with at least 100 cells analyzed in each experiment. Only cells with at least five TIFs were counted as positive. Error bars indicate s.e. (*n*=3). *P*-values were obtained using the Student’s *t*-test. ***P<0.001. (**c**) The cells were harvested for metaphase spread and FISH analysis using a telomere probe. Representative images are shown here for cells induced to KO TIN2 and TRF2. White arrows indicate chromosome fusions. Scale bars 5 μm. (**d**) Data from **c** were quantified and graphed as indicated. At least 50 metaphases were scored for each sample. White arrowheads indicate chromosome fusions. Scale bars 5 μm. *P*-values were determined by one-way analysis of variance (ANOVA). (**e**) The cells were immunoblotted using the indicated antibodies. The anti-SMC1 antibody served as a loading control. γ-irradiated Hela cells (IR+) served as positive controls. (**f**) Data from **e** were quantified. At least three experiments with independent doxycycline inductions were performed and the results were combined. Signals for phosphorylated Chk1 and Chk2 were normalized against SMC1 signals and graphed as indicated. Error bars indicate s.e. *P*-values were obtained using the Student’s *t*-test. *P<0.05, **P<0.01.

Considerable efforts have been devoted to delineating the complex signaling pathways that protect telomeres and prevent the activation of DDR. Disruption of the telosome complex can expose telomere ends to the DDR machinery and eventually lead to chromosomal abnormalities and cell cycle arrest [8]. Immunofluorescence (IF) analysis of these inducible KO cell lines supports previous findings of the importance of core telomeric proteins to telomere protection. As evidenced by the recruitment of 53BP1 to telomere dysfunction induced foci (TIFs) (Figure 3a and b), upon doxycycline-induced KO, activation of DDRs at telomeres could be observed. Except for RAP1 KO cells, which displayed minimal increase in TIFs, all other KO cell lines exhibited significant increases in 53BP1 foci that co-stained with a telomere DNA marker. In addition, KO of TRF2 and TIN2 resulted in a marked increase in telomere fusions (Figure 3c and d, Supplementary Figure S3B). These results again reinforce the notion that the six telomeric proteins have distinct roles in end protection and genomic stability.

Increased DDRs at telomeres can lead to activation of ataxia-telangiectasia mutated (ATM) and ATM- and Rad3-related (ATR) signaling, and the subsequent phosphorylation of Chk2 and Chk1, respectively. TRF2 dysfunction has been shown to activate ATM pathways [58, 59], whereas the POT1-TPP1 heterodimer is important for inhibiting ATR activation [60, 61]. Indeed, marked induction of phosphorylation of Chk2 upon TRF2 KO and Chk1 upon POT1/TPP1 KO was observed (Figure 3e and f, Supplementary Figure S4). In comparison, RAP1 deletion had no impact, whereas TPP1 and TRF1 appear to be more specific for ATR-mediated DDR regulation. TIN2 and POT1 are both important for DDR, and their KO resulted in robust phosphorylation of both Chk2 and Chk1. The Chk2 response in POT1 KO cells was somewhat unexpected, because deletion of mouse Pot1a mainly induced Chk1 activation and Pot1b inactivation mostly impacted telomere overhangs [60–65]. Perhaps Chk2 activation in our POT1 KO cells was a result of reduced telomere-associated TRF2 and TIN2 upon POT1 deletion. These results further highlight the complex mechanisms that are in place to protect telomeres from DDR and the distinct signaling events mediated by each subunit, and suggest that more functional differences may exist between human and mouse telomeric proteins in checkpoint response than previously thought.

### Telomere length maintenance and telomere overhang protection in inducible KO cells

Each of the six telomeric proteins participates in the regulation of telomere length. Previous assessment of their roles in telomere length control has mostly relied on RNAi KD and overexpression of mutant proteins in human cells, which has sometimes yielded conflicting results. For example, overexpression and RNAi experiments indicate that TPP1 and POT1 negatively regulate telomere length [21, 22, 24, 29, 66–68], but disrupting the TEL patch (TPP1 glutamate (E) and leucine (L)-rich patch) within TPP1 led to decreased telomere length [69], the latter consistent with the positive role TPP1 has in recruiting and promoting telomerase activity [25, 26, 70, 71]. In this study, we sought to better understand how inactivating individual telomeric proteins may impact telomere length control using the inducible KO cells.

**Figure 4:**
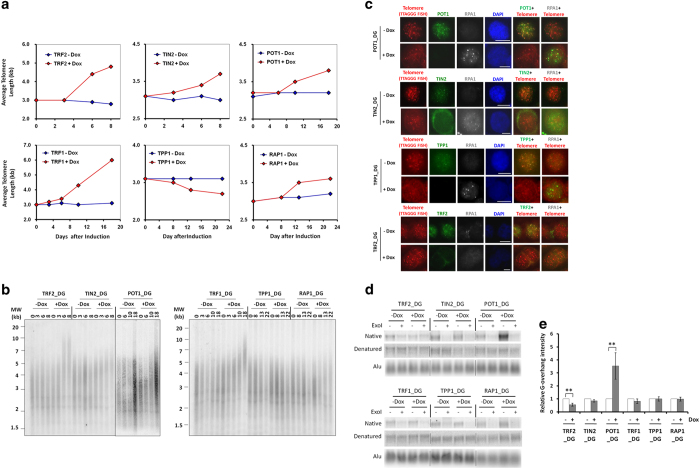
Deletion of individual subunits impacts telomere length and overhang maintenance. The inducible KO cell lines were maintained in the presence (+) or absence (−) of doxycycline (Dox) and collected at different time points for the following assays. At least three experiments with independent doxycycline inductions were performed and the results were combined. (**a**, **b**) Genomic DNA was extracted from the cells for telomere restriction fragment (TRF) analysis using a ^32^P-labeled telomere probe (TTAGGG)_3_. Telomere signals were quantified and processed using TeloRun, and average telomere length was calculated and graphed for each cell line in **a**. Representative gels of the TRF assay are shown in **b**. (**c**) The cells were collected 6 days after induction and immunostained using antibodies against each targeted protein and RPA1 along with a telomere PNA probe. 4,6-Diamidino-2-phenylindole (DAPI) was used to stain the nuclei. Three independent experiments were carried out with at least 100 cells examined for each experiment. Scale bars 10 μm. (**d**) The cells were harvested 6 days after induction for genomic DNA extraction. The DNA was then processed in the presence (+) or absence (−) of Exonuclease I (ExoI) for in-gel hybridization analysis of ss G overhangs. G overhangs were detected in the native gel using the ^32^P-labeled (CCCTAA)_3_ probe. Total telomeric DNA and Alu repeat signals were determined under denaturing conditions. (**e**) Overhang signals for each cell line from **d** were quantified and normalized against Alu repeat signals. Results from doxycycline-treated samples were compared with untreated samples and graphed as indicated. At least three independent experiments were performed for each cell line. Error bars indicate s.e. (*n*=3). *P-*values were obtained using the Student’s *t*-test. **P<0.01.

Deleting the telomere duplex binding proteins TRF1 and TRF2 resulted in significantly elongated telomeres within a few days following doxycycline addition (Figure 4a and b, Supplementary Figure S5). In the case of TRF2, increased telomere fusions following induced KO (Figure 3c and d) likely led to the apparent rapid increase in telomere length observed here. In comparison, inducible RAP1, TIN2 and POT1 KO cells showed a more gradual increase in telomere length. The TPP1 KO cells exhibited moderate acceleration of telomere shortening, which is more in line with TPP1’s role in recruiting and promoting telomerase activity.

Mammalian telomeres are thought to adopt the t-loop structure, with the ss G overhang invading into the duplex DNA [72–74]. The G overhangs are maintained through telomere DNA synthesis and active resection by exonucleases of the C-strand on 5′ ends [4, 8]. Evidence suggests that the POT1–TPP1 complex coats telomere G overhangs, inhibits nucleolytic attacks and prevents the binding of the nonspecific ssDNA binding protein RPA1 and subsequent activation of ATR-mediated checkpoint responses [29, 30, 75–78]. Furthermore, TIN2 deletion in mice also led to RPA1 accumulation and ATR activation, in line with its role in tethering POT1–TPP1 to telomeres [78]. Consistent with these previous findings, inhibition of TPP1, POT1 and TIN2 led to aberrant accumulation of RPA1 at telomeres in 13.2%, 21.7% and 12.5% of the cells, respectively (Figure 4c), indicating deprotected G overhangs. In contrast, no upregulated telomeric recruitment of RPA1 could be observed upon deletion of TRF2, TRF1 or RAP1 (Figure 4c and Supplementary Figure S6). It is possible that TRF1 and TRF2 can each independently bring the TPP1–POT1 complex to telomeres to protect G overhangs.

In mice, ablation of *Tin2*, *Tpp1* or *Pot1a/b* led to extended overhang length [48, 62, 63, 78–81]. Surprisingly, of the six KO lines, only cells induced to KO POT1 exhibited an increase in overhangs (Figure 4d and e). We found extensive chromosomal fusions upon TFR2 KO (Figure 3c and d), which likely compromised overhang protection and caused the slight decrease in G overhang length in TFR2 KO cells. Overlapping phenotypes in mouse cells knocked out of *Tin2*, *Tpp1* or *Pot1a/b*, such as TIF induction and overhang elongation, underline the interdependence of these proteins. The unexpected lack of overhang elongation in our TIN2 and TPP1 KO cells suggests that POT1 may have a protection function independent of TIN2 and TPP1 in human cells, a major difference between mouse and human cells in overhang regulation.

### Human POT1 isoforms participate in telomere overhang regulation

**Figure 5:**
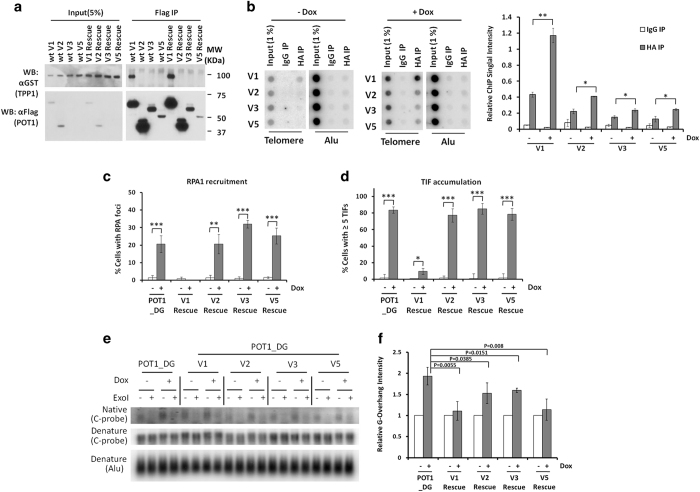
Human POT1 variants have important roles in maintaining telomere overhangs. (**a**) 293T cells transiently co-expressing GST-tagged TPP1 and Flag-tagged human POT1 variants were immunoprecipitated (IP) with anti-Flag antibodies and blotted as indicated. In addition to wild-type (wt) POT1 variant proteins, rescue constructs encoding POT1 variants that contained sgRNA-resistant silent mutations (rescue) were also examined. POT1 KO cells stably expressing the sgRNA-resistant rescue constructs were then induced with doxycycline for 6 days before being harvested for the assays described below. (**b**) For telomere ChIP analysis, the rescue cells were immunoprecipitated (IP) with anti-HA antibodies to bring down associated telomeric DNA for dot-blotting and hybridization with a telomere repeat probe (TTAGGG)_3_ (left panel). IgG was used as a negative control. Telomeric signals were quantified and normalized against Alu repeat signals and graphed on the right. Error bars indicate s.e. *P*-values were obtained using one-way analysis of variance (ANOVA). *P<0.05, **P<0.01. (**c**) To assess RPA1 recruitment to telomeres, cells were immunostained with antibodies against RPA1 along with a telomere PNA probe. The data were quantified and graphed as shown. Three independent experiments were carried out for each cell line with at least 100 cells in each experiment. Error bars indicate s.e. (*n*=3). *P*-values were obtained using the Student’s *t*-test. **P<0.01, ***P<0.001. (**d**) To assess possible changes in TIFs, cells were immunostained using antibodies against 53BP1 along with a telomere PNA probe. The data were quantified and graphed as shown. Three independent experiments were carried out for each cell line with at least 100 cells in each experiment. Only cells with at least five TIFs were counted as positive. Error bars indicate s.e. (*n*=3). *P*-values were obtained using the Student’s *t*-test. *P<0.05, ***P<0.001. (**e**) Genomic DNA was extracted from the cells for processing in the presence (+) or absence (−) of Exonuclease I (ExoI) before hybridization analysis of ss G overhangs in the native gel using the (CCCTAA)_3_ probe. Total telomeric DNA and genomic DNA (Alu) signals were determined under denaturing conditions. (**f**) Overhang signals from **e** were quantified and normalized against Alu repeat signals and graphed as indicated. At least three independent experiments were performed for each cell line. Error bars indicate s.e. (*n*=3). *P*-values were calculated using one-way analysis of variance (ANOVA).

The KO cell lines offer a unique opportunity for us to investigate the possible functional significance of splicing variants of telosome subunits. Although human has one POT1 gene as opposed to two in mice, a total of five alternatively spliced forms of hPOT1 have been described to date [15]. hPOT1 V1 is the full-length form that has been extensively studied (Supplementary Figure S7A). Little is known about the functional significance of the other isoforms, which appear to be expressed in normal and cancer tissues as well as cancer cell lines [15, 82]. The V4 variant was not examined here because POT1 coding sequences are interrupted by an early stop codon. The remaining isoforms share with V1 the N-terminal OB-fold domain but differ in their C-termini. We have designed the dual sgRNAs to inactivate all of the POT1 isoforms (Supplementary Figure S7A), enabling us to determine the role of each isoform individually. We expressed CRISPR-resistant POT1 V1 isoform in the POT1 KO cells and examined the cells for overhang status, TIF formation and RPA1 accumulation (Figure 5 and Supplementary Figure S7B–D). As expected, POT1 V1 could localize to telomeres (Figure 5b), and rescue the phenotypes of increased TIFs, accumulated RPA1, and excessively long overhangs (Figure 5c–f). These data support V1 as the main POT1 isoform that caps telomere ends and shields them from DDRs.

Consistent with their lack of TPP1-interacting domains, POT1 variants V2, V3 and V5 could not interact with TPP1 (Figure 5a). All three isoforms do contain intact OB-folds that can mediate telomere ssDNA binding, and therefore may target to telomeres independent of TPP1. In support of this notion, our telomere ChIP experiments showed that POT1 V2, V3 and V5 could indeed associate with telomeres (Figure 5b and Supplementary Figure S7B), albeit with markedly reduced abilities compared with V1. Binding of POT1 isoforms to telomeres appeared to increase in the absence of endogenous POT1 (Figure 5b), probably because of more sites becoming available upon POT1 deletion, or because of increased overhang length.

Individual expression of the short POT1 isoforms in POT1 KO cells could not rescue the phenotypes of RPA1 accumulation or increased telomere DNA damage (Figure 5c and d and Supplementary Figure S7C and D). However, they consistently reduced the increase in telomere overhangs in these cells, albeit to varying degrees (Figure 5e and f). These data indicate that the shorter human POT1 isoforms can regulate overhang length independent of TPP1. Furthermore, this previously unknown function of the POT1 isoforms may help to explain some of the differential phenotypes observed between mouse and human KO cells.

### Deletion of telosome subunits leads to metabolic perturbations in the inducible KO cells

Complete deletion of essential genes such as *TRF2* and *TIN2* causes cell cycle arrest and/or death, making it difficult to isolate single KO clones or obtain large numbers of cells for extensive biochemical studies. Our inducible KO system bypasses the need of KO cell cloning, and enables the expansion of cells to large quantities before KO induction for biochemical analysis such as metabolomic profiling.

**Figure 6:**
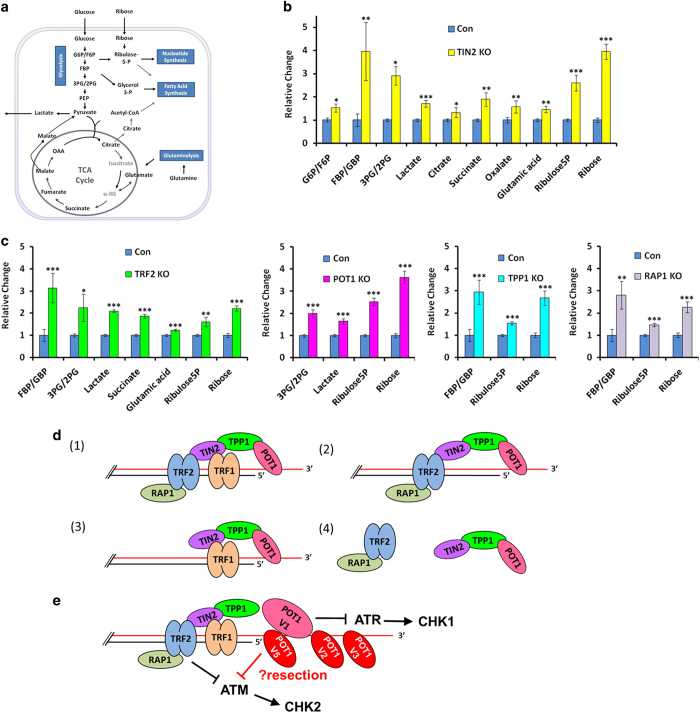
Deletion of individual subunits impacts metabolic pathways in the inducible KO cells. (**a**) Core metabolic pathways and key metabolites in mammalian cells are highlighted. G6P, glucose-6-phosphate. F6P, fructose-6-phosphate. FBP, fructose-1,6,-biphosphate. 3PG/2PG, 3-phosphoglycerate/2-phsphoglycerate. PEP, phosphoenolpyruvate. OAA, oxaloacetate. (**b**,** c**) Inducible KO cells were cultured in the presence of doxycycline for 6 days before being collected in replicates and processed for targeted metabolomic analysis by LC-MS. Cas9-inducible parental cells were grown in the presence of doxycycline and used as controls (Con). The results were normalized to internal standards and metabolites that consistently showed differences in two experiments with independent doxycycline inductions are presented here. Error bars correspond to s.d. of three independent experiments. *P-*values were calculated using one-way analysis of variance (ANOVA). *P<0.05; **P<0.01; ***P<0.001. (**d**) The six telomere proteins may assemble and function on telomeres as a single unit (1), a TRF1-less five-protein subcomplex (2), or a four-protein subcomplex without TRF2/RAP1 (3). Non-telomere bound subcomplexes also exist (4). (**e**) A model of telomere protection that incorporates human POT1 isoforms highlights the unique features of the human system.

Crosstalk between telomere maintenance and metabolic pathways has been well documented, with several key telomere regulators including TERT and TIN2 implicated in more direct metabolic regulation [33–37, 83]. We therefore examined TIN2 KO cells and determined how completely disrupting TIN2 affected metabolic control, especially with respect to key metabolites in glycolysis and the tricarboxylic acid (TCA) cycle. Research has shown that cancer cells often consume large quantities of glucose, which fuels the TCA cycle, as well as pathways for macromolecule synthesis (for example, nucleotides, amino acids and lipids) (Figure 6a) [84, 85]. Glucose and other metabolites such as glutamine serve as substrates in various bioenergetic pathways to support growth of cancer cells in which upregulated glycolysis and glutaminolysis pathways have often been found.

Given that treatment of cells with drugs such as doxycycline can drastically alter cellular metabolomes, we decided to compare doxycycline-induced TIN2 KO cells with doxycycline-treated Cas9-inducible parental cells that did not express any sgRNA sequences. The inducible TIN2 KO cells were expanded and then treated with doxycycline for 6 days before being collected for analysis by quantitative liquid chromatography—mass spectrometry (LC-MS). As shown in Figure 6b, TIN2 KO led to varying changes in a broad range of metabolites in glycolysis, TCA cycle and macromolecule synthesis. When we examined the other telosome subunits, we found TRF1 KO to have the least impact, only consistent increases in ribose (data not shown). In comparison, TRF2 KO led to reproducible increases in a number of metabolites (Figure 6c). Similarly, knocking down the remaining subunits resulted in reproducible and differential changes in certain key metabolites in glycolysis and macromolecule synthesis. These findings reaffirm the distinct roles that each subunit has in ensuring the growth and proliferation of the cell. None of the other proteins examined had the same widespread effect on metabolism as TIN2. For example, TIN2 was the only telosome subunit whose KO affected multiple metabolites in the TCA cycle, which occurs in the mitochondria. This observation supports our previous findings that TIN2 can localize to the mitochondria and regulate the metabolic pathways in the mitochondria.

## Discussion

Work using mouse models, mutant proteins and RNAi to probe the functional significance of the telosome and its subunits has greatly advanced our knowledge and understanding of telomere homeostasis. Genetically modified mouse models have been indispensable to loss-of-function studies, but differences between mouse and human, as well as the cost and efforts associated with mouse studies continue to pose challenges. Major drawbacks of RNAi-mediated KD include its off-target effects and the inability to achieve complete inhibition. As a result, many questions regarding human telomere maintenance remain unanswered. The RNA-guided CRISPR/Cas9 genome-editing technology has enabled unprecedented manipulations of the genome in a much more targeted and efficient manner, particularly in somatic cells and cell lines [39–47]. In this report, we describe the systematic generation and profiling of inducible KO cell lines for the six core telomere proteins. In all of the experiments presented, the results came from multiple independent doxycycline inductions of the inducible KO cells. Such reproducibility and consistence underscore the robustness of the inducible system and the advantage of using polyclonal populations in cellular assays. We can now more clearly define the function of human telomeric proteins and identify differential regulatory mechanisms in human vs mouse.

### Organization of the human telosome/shelterin complex

With the inducible KO system, we now have a clearer picture of how the human telosome complex may be organized (Figure 6c). Of the six subunits, RAP1 only interacts with TRF2. Except for telomere length changes, induced RAP1 deletion had no major impact in most of the telomere assays described here, consistent with previous analyses using RAP1-inactivated mouse and cellular models [86–88]. In addition, removing either TIN2, TPP1 or POT1 markedly impacted the telomere targeting of the other two proteins, providing strong evidence that these three proteins likely form a functional unit on telomeres. Interestingly, TRF2 KO affected the telomeric binding of all the other subunits except for TRF1, supporting the existence of the five-protein TRF1-less complex (Figure 6d).

### The role of POT1 in G overhang protection

In mice, both Pot1a and Pot1b can bind Tpp1 and are tethered to telomeres through Tpp1-Tin2; however, the two mouse POT1 proteins participate in distinct signaling events for telomere regulation [68, 89]. Human POT1, in comparison, appears to carry out the functions attributed to both POT1a and POT1b. It is therefore expected that depletion of TPP1 or TIN2 in human cells would disrupt POT1 targeting to telomeres and POT1-mediated protection and length regulation of G overhangs. For example, *Tpp1* KO MEFs showed similar phenotypes as cells doubly knocked out for Pot1a/b [79, 80]. Although POT1 KO in our inducible cells led to expected increases in overhang length and RPA1 staining, we were surprised to discover a lack of accumulation of elongated telomere ssDNA in cells knocked out of TIN2 or TPP1. Of the four splicing variants of human POT1 examined in this study, only V1 can interact with TPP1, likely because it is the only variant that retains the C-terminal TPP1-interacting domain (Figure 5a and Supplementary Figure S7A). Although isoforms V2, V3 and V5 do not bind TPP1, they can still associate with telomeres (Figure 5a and b). Given that our POT1 KO strategy disrupts isoforms V2, V3 and V5 as well, we speculate that these POT1 isoforms may participate in overhang protection independent of TPP1 and that the unexpected results seen in TIN2 and TPP1 KO cells help to highlight this shared function between different human POT1 isoforms (Figure 6e).

Based on this model, telomere targeting of the full-length variant POT1 V1 is disrupted in TPP1 and TIN2 KO cells; however, telomere ssDNA overhangs can still be protected by other variants, which can localize to telomeres independent of TPP1-TIN2. These OB-fold only POT1 proteins may associate with telomeres directly or through interaction with other OB-fold containing proteins. Indeed, when we ectopically expressed V2, V3 or V5 in the POT1 KO cells, they could rescue the overhang length phenotype to varying degrees. Similar findings were previously reported for POT1 V5 in POT1 KD cells [82]. Interestingly, POT1 V2, V3 and V5 could not rescue the TIF or RPA1 phenotypes of POT1 KO cells, suggesting distinct pathways for different POT1 isoforms in regulating overhang length vs DDR. It is possible that once V2, V3 and V5 are recruited to telomeres, they can block exonucleases such as Exo1 from further recessing the 3’ end of telomeres. Collectively, our study supports a new model of both TPP1-dependent and -independent regulation of telomere overhangs by human POT1, in contrast to the TPP1-dependent model for mouse Pot1a/b.

### The role of telomeric proteins in metabolic control

Although previous studies have implicated telomeric proteins in metabolic regulation, this is the first time that metabolic changes were systematically investigated upon deletion of individual subunits of the shelterin/telosome complex. It is possible that the metabolic alterations observed in our KO cells were indirect results of activation of DDR pathways and changes in telomere length. However, the differences in the metabolomes in these cells suggest distinct and significant impact on cellular metabolism as a result of inhibition of different human telomeric proteins. For instance, TIN2 KO appeared to impact many of the metabolites in different metabolic pathways, including the TCA cycle. The TCA cycle, which occurs in the mitochondria where it metabolizes the end products of glycolysis and feeds into oxidative phosphorylation, is central to energy production and biosynthesis. The finding that only TIN2 KO appeared to impact TCA cycle metabolites supports our previous findings of TIN2 targeting to the mitochondria, and is consistent with idea that TIN2 can directly regulate metabolism. Interestingly, although TRF2 KO appeared to also affect multiple glycolytic, glutaminolytic and nucleic acid synthesis intermediates, knocking out the remaining subunits was more restricted in terms of changes in the metabolome. Whether such differences are linked to the predominant function of TRF2 in ATM-mediated DDR response warrants further investigation. Taken together, these data underline the complex crosstalk between telomere maintenance and metabolic control.

### Application of the inducible CRISPR/Cas9 KO system

For genes essential for growth and survival, the inducible KO cell lines afford the time window needed to carry out biochemical studies before the cells undergo growth arrest. For example, we could not obtain straight KO clones of cells deleted for TIN2, TRF2 or POT1, but the inducible KO cells have allowed us to explore the functions of these proteins in a variety of assays. In theory, a single sgRNA targeting a specific site within a locus should effectively generate cells with frame-shift indels that inactivate the target gene. In our induced single sgRNA-targeted KO cells, we often found low expression levels of the target genes after Cas9 induction. This residual expression is likely a result of the induced KO cells containing a mixture of alleles with different indels (some of which cannot completely disrupt target gene function), and differs from that observed in RNAi KD cells. In the latter, every cell likely still expresses the target gene at a certain level following incomplete suppression by small interfering RNAs/short hairpin RNAs. The polyclonal induced KO cells, on the other hand, comprise mostly of cells completely knocked out for the target gene, with a small fraction of cells that may contain in-frame indels or can ‘restore’ expression following extended culturing. This is an important distinction because residual expression in nearly every RNAi KD cell may be sufficient for the entire population to ‘behave’ normally in an assay. However, a very small fraction of cells with heterozygous KO or wild-type alleles are unlikely to dilute or mask the response of the whole population, if the overwhelming majority of cells have no expression of the gene.

Based on our data, more complete deletion and inactivation can be achieved with two sgRNAs. A second sgRNA significantly reduced the possibility of in-frame ligations, as well as the ability of cells to overcome inactivating mutations, although with the caveat of possibly increasing off-targets. As the induced cells are not single clones, possible complications in data interpretation from off-target effects may be less likely. Moreover, expression of CRISPR-resistant constructs could rescue the observed phenotypes, which helped to rule out potential off-target effects.

It appeared to take similar amount of time for our inducible cells to achieve efficient KO as for cells transfected with plasmids encoding Cas9 and sgRNA to generate straight KO. The isolation of straight KO cells, however, requires significantly longer time (for non-essential genes), whereas large numbers of induced KO cells can be more quickly obtained. The inherently mixed nature of the induced KO population does have the potential to introduce variations. The distribution of various populations (+/+ vs +/− vs −/−) should in theory be similar each time the cells are induced. Deep sequencing of the induced KO cells may help to shed light on the exact dynamics of the KO populations, but the short reads of such methods will fail to capture any large deletions, especially with our dual sgRNA cells. Any variability in population dynamics is more likely to be caused by variable Cas9 induction than the constitutively expressed sgRNAs. Hence, using the same inducible Cas9 clone to generate all six cell lines for comparative studies should help minimize variations. Furthermore, each of our cell lines had been induced multiple times independently for each assay and the results were found to be similar, attesting to the reproducibility of the system.

The six inducible cell lines should prove particularly useful to investigators who may be interested in studying different aspects of telomere maintenance. For instance, in addition to the ability to assay cells in which essential genes are deleted, this system also enables real-time comparisons of specific protein complexes before and after the removal of a key subunit. Furthermore, our method makes possible both the production of large numbers of genetically edited cells for studies of essential genes and the generation of more well-defined snapshots of cells in response to telomere perturbations.

## Materials and methods

### Vector construction

Sequences encoding the humanized Cas9 gene under the control of the tetracycline-responsive promoter were cloned into a lentiviral vector that also encodes rtTA [90]. Individual sgRNA sequences (Supplementary Table S1) were cloned into modified vectors encoding different antibiotic resistance genes (puromycin, blasticidin or hygromycin). These vectors are based on the LentiCRISPR vector (GeCKO) but no longer contain Cas9 sequences [90]. Complementary DNAs encoding wild-type and rescue mutants for sgRNA-resistant hTIN2 and hPOT1 isoforms were cloned into a pHAGE-based lentiviral vector for C-terminal tagging with HA and FLAG epitopes [91]. Rescue constructs were generated by introducing either single-nucleotide mutations in the PAM sequence (TIN2 sgRNA1: GGG→ GAG, TIN2 sgRNA2: TGG→ TGA, POT1 sgRNA1: AGG→ AGA) or double-nucleotide mutations in the sgRNA target region (POT1 sgRNA2: GGAGGTACCAGTTACGGTCG→ GGAGGTACCAGTTACGGAAG).

### Generation of inducible CRISPR KO cell lines

Hela cells stably expressing doxycycline-inducible Cas9 were first generated by lentiviral transduction. A single clone with robust and efficient Cas9 induction was selected for further experiments. Vectors expressing single or dual sgRNAs were stably introduced into the Cas9-inducible cells by lentiviral transduction followed by selection with appropriate antibiotics. The appropriate concentrations for Cas9 induction and efficient cleavage at the intended locus were determined for each cell line. We have found that 6 days of incubation in 1 μgml^−1^ of doxycycline is optimal for our cells. Successful inactivation of each gene was confirmed by western blotting with the appropriate antibodies. Further validation was conducted by extracting genomic DNA from the cells either for direct sequencing or for TOPO cloning before Sanger sequencing.

### Immunoprecipitation, immunoblotting and telomere ChIP assays

Co-immunoprecipitation studies were performed as described previously [19]. Cells were lysed in 1×NETN buffer (1 m Tris-HCl (pH 8.0), 1 mM EDTA, 100 mM NaCl and 0.5% Nonidet P-40) containing 1 mM DTT and a proteinase inhibitor mixture (Roche Applied Science, Mannheim, Germany). The lysates were then immunoprecipitated with appropriate antibodies for sodium dodecyl sulfate–polyacrylamide gel electrophoresis and western blotting.

Telomere ChIP assays were performed as described previously with slight modifications [92]. Briefly, cells were chemically crosslinked in1% formaldehyde in phosphate-buffered saline, and sonicated to shear chromatin. Sonicated lysates were pre-cleared before being incubated with 3 μg of antibodies for immunoprecipitation. The co-precipitated DNA was eluted and analyzed by dot-blot and southern hybridization using the ^32^P-labeled telomere (TTAGGG)_3_ and Alu repeat probes.

Antibodies used for immunoprecipitation and western blot analyses in this study are: horseradish peroxidase-conjugated anti-glutathione S-transferase (GST) polyclonal antibody (GE Healthcare Life Science, Pittsburgh, PA, USA), horseradish peroxidase-conjugated anti-FLAG M2 antibody and M2-conjugated agarose beads (Sigma, St Louis, MO, USA), rabbit anti-FLAG polyclonal antibody (Sigma), goat anti-actin polyclonal antibody (Santa Cruz Biotechnology, Dallas, TX, USA) and rabbit anti-SMC1 antibody (Bethyl Laboratories, Montgometry, TX, USA), mouse anti-TRF2 monoclonal antibody (Calbiochem, San Diego, CA, USA), rabbit anti-RAP1 polyclonal antibody (Bethyl Laboratories), rabbit anti-POT1 polyclonal antibody (Novus Biologicals, Littleton, CO, USA), rabbit anti-TPP1 and anti-TIN2 polyclonal antibodies [6], and goat anti-TRF1 antibody [23], rabbit anti-p-Chk1(Ser317) and anti-Chk1 antibodies (Cell Signaling Technology, Danvers, MA, USA), and rabbit anti-p-Chk2(Thr68) and anti-Chk2 antibodies (Cell Signaling), mouse anti-p-ATM (Ser1981) and rabbit anti-ATM antibodies (Cell Signaling), rabbit anti-p-ATR(Ser428) and anti-ATR antibodies (Cell Signaling) and rabbit anti-HA antibody (Santa Cruz Biotechnology).

### Cell proliferation assay and cell cycle analysis

Cells were plated in 12-well plates at 1×10^4^ cells per well and maintained for 10 days with or without 1 μgml^−1^ doxycycline. The number of viable cells at various time points was determined by Trypan blue exclusion. To determine DNA content, 1×10^6^ cells maintained with or without doxycycline (1 μgml^−1^) for 6 days were collected, washed with 1×phosphate-buffered saline and then fixed in 70% ethanol at room temperature for 30 min. The fixed cells were then incubated in 0.5 ml 1×phosphate-buffered saline containing 50 μgml^−1^ propidium iodide and 0.2 mgml^−1^ DNase-free RNase A (pH 7.4) at 37 °C for 30 min. The cells were subsequently analyzed using an LSRII flow cytometry analyzer (BD Biosciences, San Jose, CA, USA).

### IF and telomere fluorescence *in situ* hybridization (FISH) analysis

IF was performed as previously described [25]. Briefly, cells grown on glass coverslips were permeabilized for 30 s with 0.2% Triton X-100, fixed in 4% paraformaldehyde and then permeabilized again with 0.5% Triton X-100, before being blocked in 5% bovine serum albumin. Cells were subsequently incubated with appropriate antibodies and/or a telomere peptide nucleic acid (PNA) probe (Bio-PNA). 4,6-Diamidino-2-phenylindole was used to visualize the nuclei. For TIF assays, >100 cells were examined for each experiment, and cells with >5 co-stained foci were counted as being TIF positive. At least three independent experiments were performed for each cell line.

Antibodies used for IF are: mouse anti-FLAG M2 and rabbit anti-FLAG polyclonal antibodies (Sigma), rabbit anti-53BP1 (NB100-304; Novus Biologicals) and mouse anti-53BP1 (BD Biosciences) antibodies, rat anti-RPA1 polyclonal antibody (Cell Signaling), goat anti-TRF1 polyclonal antibody, mouse anti-TRF2 monoclonal antibody (Calbiochem), rabbit anti-RAP1 polyclonal antibody (Bethyl Laboratories), rabbit anti-TPP1 and anti-TIN2 polyclonal antibodies [6], rabbit anti-POT1 polyclonal antibody (Bethyl Laboratories) and rabbit anti-HA antibody (Santa Cruz Biotechnology).

Metaphase spread and telomere fluorescence *in situ* hybridization analysis was performed as previously described [93]. Briefly, cells were incubated with 0.1 μgml^−1^ colcemid (KaryoMax, Invitrogen, Carlsbad, CA, USA) for 3 h and harvested. The cells were then incubated in hypotonic solution (0.075 m KCl) for 25 min at room temperature, fixed in methanol/glacial acetic acid (3:1) solution for 5 min and spread onto clean slides. The slides were treated with pepsin, fixed in 4% formaldehyde, dehydrated in successive ethanol baths (70, 90, and 100%) for 5 min each and air dried. The slides were subsequently denatured at 80 °C for 3 min and then hybridized with telomere PNA probes (Bio-PNA) at 25 °C for 2 h in the dark. The slides were then washed, dehydrated and mounted with VectaShield mounting medium containing 4,6-diamidino-2-phenylindole (Vector Laboratories, Burlingame, CA, USA). At least 50 metaphase spreads were captured using a Zeiss Imager Z1 microscope (Göttingen, Germany) and analyzed using AxioVision 4.8(Göttingen, Germany).

### TRF assay and telomere overhang analysis

Cells were first induced with doxycycline for 6 days, and then collected for TRF analysis at various time points to estimate the average length of telomeres using TeloRun [6, 94]. The in-gel detection of telomere ssDNA overhangs was performed as previously described with slight modifications [95]. Genomic DNA was digested with* Hin*fI and *Rsa*I (New England Biolabs, Ipswich, MA, USA) for 16 h. In all, 5 μg each of the digested DNA was then incubated with and without 20 U of Exonuclease I (New England Biolabs) for 6 h. The reaction mixtures were fractionated on a 1.2% agarose gel in 1× Tris/borate/EDATA (TBE) buffer for 1.5 h at 60 V and dried on a gel dryer at 50 °C for 2 h. The dried gel was pre-hybridized in hybridization buffer (0.5 m Na_2_HPO_4_ pH 7.2, 1 mM EDTA, 7% sodium dodecyl sulfate) and hybridized in fresh buffer with a ^32^P-labeled C-strand probe (CCCTAA)_3_. The gel was washed 3× with 2× SSC containing 0.1% sodium dodecyl sulfate for 30 min at room temperature and analyzed on a PhosphorImager (GE Healthcare Life Science). The gel was subsequently denatured (0.5 m NaOH, 1.5 m NaCl for 30 min), neutralized (0.5 MTris-HCl for 30 min), and then hybridized with the C-strand or Alu repeat probes as control.

### Metabolomic analysis by liquid chromatography-mass spectrometry

Each cell line was induced with 1 μgml^−1^ doxycycline, cultured and harvested in multiple (3–4) replicates (~5×10^6^ cells each), and frozen in aliquots before metabolome extraction as described previously [96]. Briefly, cells were three times frozen and thawed and resuspended in ice-cold methanol:water (750 μl, 4:1) containing 20 μl of internal standards (Tryptophan-15N2, Glutamic acid-d5, Thymine-d4, Gibberellic acid, Trans-Zeatin, Jasmonic acid, Anthranilic acid and Testosterone-d3, all from Sigma-Aldrich). Homogenization entailed 2×30-s pulses, 10-min vortex mixing with ice-cold chloroform (450 μl), and 2-min vortex mixing with ice-cold water (150 μl). The homogenate was incubated at −20 °C for 20 min and centrifuged at 4 °C for 10 min to partition aqueous and organic layers for drying at 37 °C for 45 min. The aqueous extract was reconstituted in 500 μl of ice-cold methanol:water (50:50) and filtered at 4 °C for 90 min through 3 kDa molecular filters (AmiconUltracel −3 K Membrane, Millipore Corporation, Billerica, MA, USA). The filtrate was dried for 45 min at 37 °C before resuspension in 100 μl of methanol:water (50:50) containing 0.1% formic acid (Sigma-Aldrich). High-performance liquid chromatography (HPLC) analysis was performed using an Agilent 1290 series HPLC system equipped with a degasser, binary pump, thermostatted autosampler and column oven (Agilent Technologies, Santa Clara, CA, USA). The multiple reaction monitoring-based measurement of relative metabolite levels used reverse or normal phase chromatographic separation. All samples were kept at 4 °C and 5 μl was used for analysis.

TCA metabolites were separated through normal phase chromatography. The binary pump flow rate was 0.2 mlmin^−1^ with 80% B to 2% B gradient over 20 min, 2% B to 80% B for 5 min and 80% B for 13 min. The flow rate was gradually increased as follows: 0.2 mlmin^−1^ (0–20 min), 0.3 mlmin^−1^ (20.1–25 min), 0.35 mlmin^−1^ (25–30 min), 0.4 mlmin^−1^ (30–37.99 min) and 0.2 mlmin^−1^ (5 min). Metabolites were separated on a Luna Amino (NH2) column (4 μm, 100A 2.1×150 mm, Phenominex, Torrance, CA, USA) in a temperature-controlled chamber (37 °C). All columns used were washed and reconditioned after every 50 injections. HPLC-grade acetonitrile, methanol and water were from Burdick & Jackson (Morristown, NJ, USA). The calibration solution containing multiple calibrants in acetonitrile/trifluroacetic acid/water was from Agilent Technologies. Data were curated through quality control assessment (using data from sample pools) and normalization using internal standards.

### Statistical analysis

All experiments were independently repeated at least three times and presented as mean±s.d. or mean±s.e. Statistical analyses were performed using either Student’s *t*-test or one-way analysis of variance. Significant differences were defined as *P*<0.05 or lower.
