# Supplementary material for: Systematic analysis of human telomeric dysfunction using inducible telosome/shelterin CRISPR/Cas9 knockout cells
Source: Cell Discov. 2017 Sep 26;3:17034–. doi: 10.1038/celldisc.2017.34 (PMC5613224; doi:10.1038/celldisc.2017.34)
Supplement: Supplementary Information [file celldisc201734-s1.pdf]

Supplementary Information

Supplementary Figure S1. The tetracycline-inducible CRISPR system. **A)** Hela cells stably expressing shRNA sequences against TRF2 were examined by western blotting. shCon, shRNA sequence against GFP served as a negative control. **B)** Of the two-vector inducible CRISPR/Cas9 system, one lentiviral vector encodes Cas9 under the tetracycline-inducible promoter and the *tet*-controlled transactivator (rtTA), and the other lentiviral vector encodes the guide RNA sequence. **C)** Western blotting analysis of the Hela cell clone with robust and dose-dependent expression of Cas9 in response to different concentrations of doxycycline (dox). Actin served as a loading control.

Supplementary Figure S2. Generation of inducible KO cell lines for the six core telomere proteins. A) The region encompassing the sgRNA target sites was PCR amplified for TOPO cloning and Sanger sequencing for TIN2_DG cells. All of the 20 TOPO clones sampled are consistent with TIN2 inactivation, with most clones containing large deletions of the intervening sequences while the remainder having small indels at both target sites. **B)** Positions of sgRNA target sites within the genomic loci of the six telomeric proteins are indicated. **C)** Knockout efficiencies of single vs. dual (DG) sgRNA strategies are compared. Cells were collected at different days after dox induction for immunoblotting with the appropriate antibodies. Antibodies against SMC1 and actin were used as loading controls. Molecular weight makers (kDa) are indicated on the right.

Supplementary Figure S3. Analysis of inducible KO cell lines. A) Cells were collected six days after Dox induction for telomere ChIP assays using appropriate antibodies and dot blots

with a $(TTAGGG)_3$ probe. IgG served as a negative control. **B)** Cells were harvested for metaphase spread and FISH analysis using a telomere probe. Data were quantified and graphed as indicated. At least 50 metaphases were scored for each sample.

Supplementary Figure S4. Activation of ATM and ATR in individual KO cell lines. Cells were immunoblotted using the indicated antibodies. The anti-SMC1 antibody served as a loading control.

Supplementary Figure S5. Genomic DNA was extracted at the indicated time points following Dox induction from various KO cells for hybridization using a ^{32}P-labeled Alu repeat probe, in addition to analysis using the telomere probe $(TTAGGG)_3$ (Figure 4B).

Supplementary Figure S6. Knockout of RAP1 and TRF1 had no effect on the telomeric recruitment of RPA1. Cells were collected six days after induction and immunostained using antibodies against RAP1 or TRF1 and RPA1 along with a telomere PNA probe. DAPI was used to stain the nuclei. Scale bars 10 µm

Supplementary Figure S7. Analysis of POT1 variants in POT1 KO cells. A) Genomic organization of the five splice variants of human POT1. Exons that are added (12a and 15a) or skipped (8 and 17) in various isoforms are in yellow. **B)** POT1 KO cells ectopically expressing different HA-tagged POT1 variants (that were also sgRNA resistant) were collected 7 days after Dox induction, and blotted with the antibodies indicated. Antibodies against SMC1 served as loading controls. * indicates the expected size for the exogenously expressed POT1 variant

proteins. **C)** POT1 KO cells ectopically expressing different POT1 variants (that were also sgRNA resistant) were stained 6 days after dox induction with anti-RPA1 antibodies and a telomere PNA probe. DAPI was used to stain the nuclei. Scale bars 10 μm **D)** POT1 KO cells ectopically expressing different HA-tagged POT1 variants (that were also sgRNA resistant) were stained six days after dox induction with the indicated antibodies and a telomere PNA probe. DAPI was used to stain the nuclei. Scale bars 10 μm

Supplementary Table S1. List of gRNAs, shRNAs, and primers used in the study.

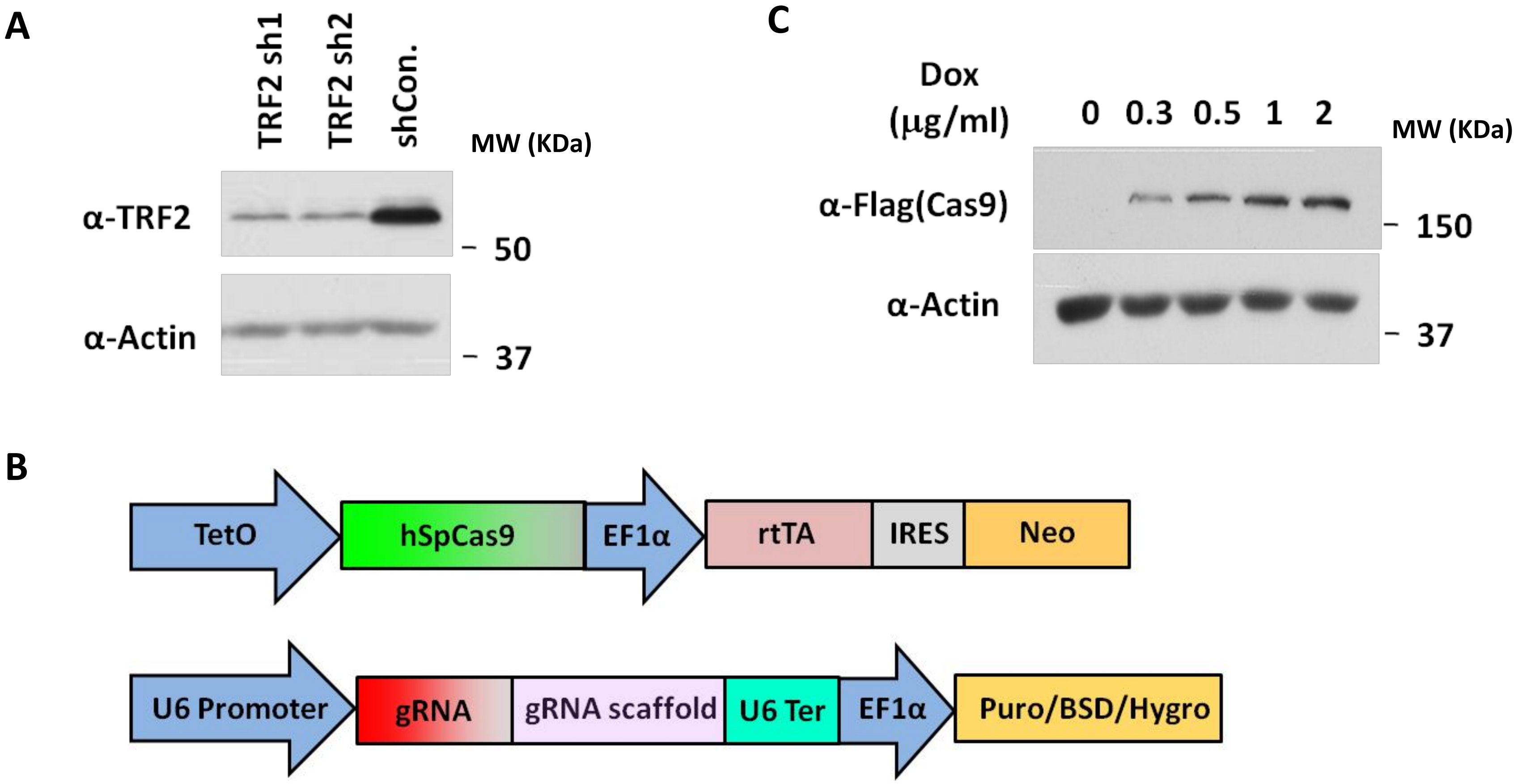
A
TRF2 sh1
TRF2 sh2
shCon.
MW (KDa)
α-TRF2
50
α-Actin
37
C
Dox
(μg/ml)
0
0.3
0.5
1
2
MW (KDa)
α-Flag(Cas9)
150
α-Actin
37
B
TetO
hSpCas9
EF1α
rtTA
IRES
Neo
U6 Promoter
gRNA
gRNA scaffold
U6 Ter
EF1α
Puro/BSD/Hygro

A

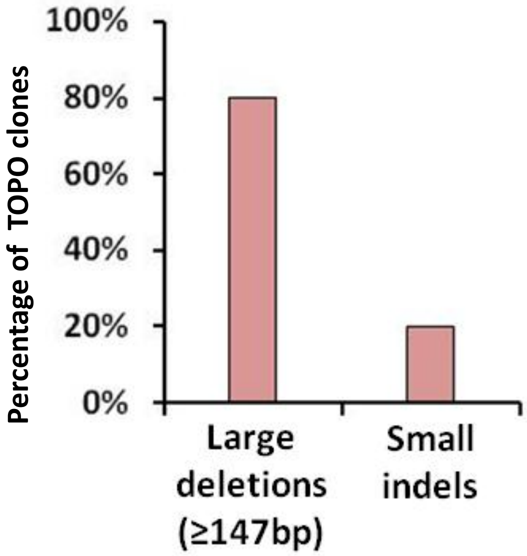
Percentage of TOPO clones
100%
80%
60%
40%
20%
0%
Large deletions (≥147bp)
Small indels

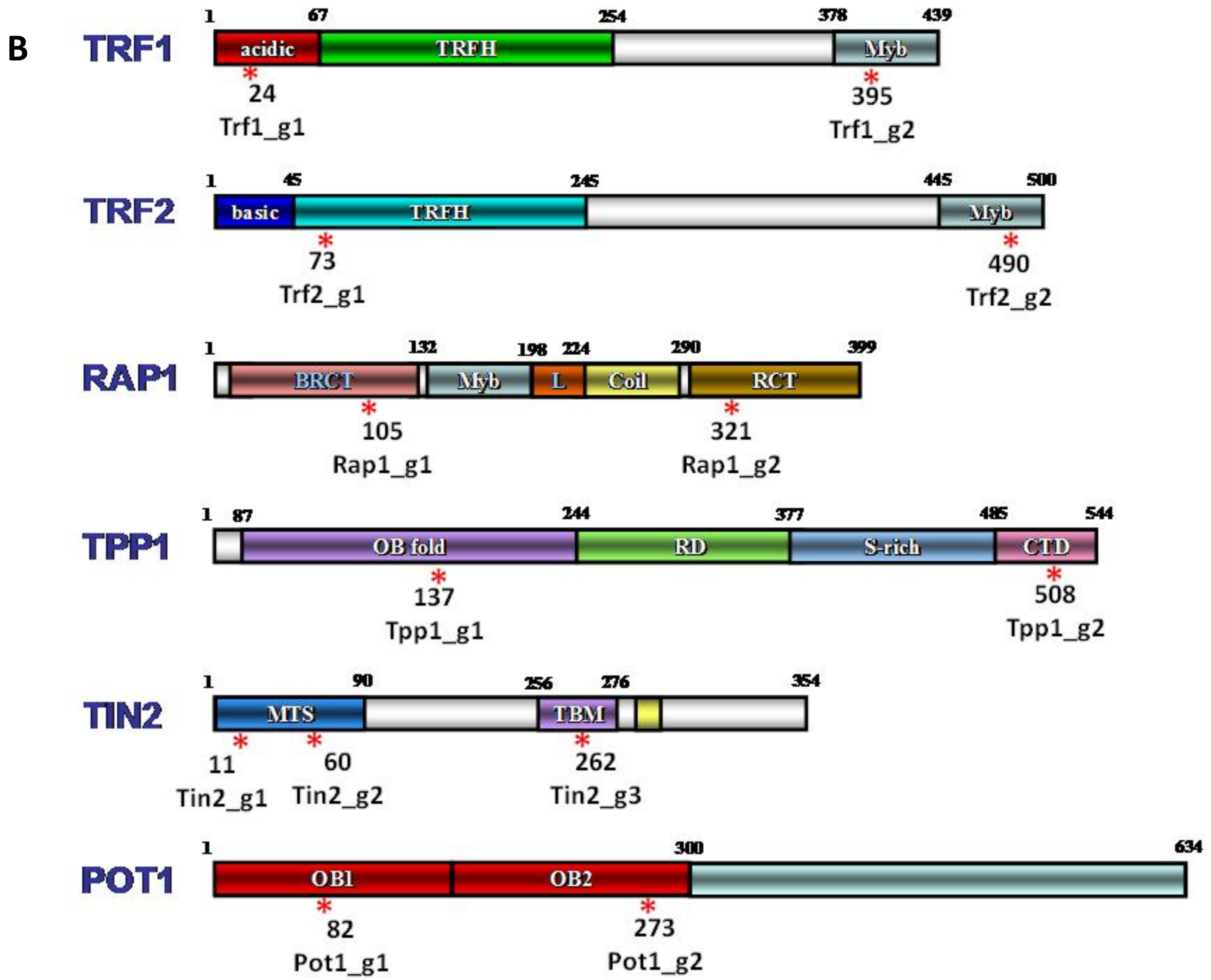
B
TRF1
1
67
254
378
439
acidic
TRFH
Myb
24
Trf1_g1
395
Trf1_g2
TRF2
1
45
245
445
500
basic
TRFH
Myb
73
Trf2_g1
490
Trf2_g2
RAP1
1
132
198
224
290
399
BRCT
Myb
L
Coil
RCT
105
Rap1_g1
321
Rap1_g2
TPP1
1
87
244
377
485
544
OB fold
RD
S-rich
CTD
137
Tpp1_g1
508
Tpp1_g2
TIN2
1
90
256
276
354
MTS
TBM
11
Tin2_g1
60
Tin2_g2
262
Tin2_g3
POT1
1
300
634
OB1
OB2
82
Pot1_g1
273
Pot1_g2

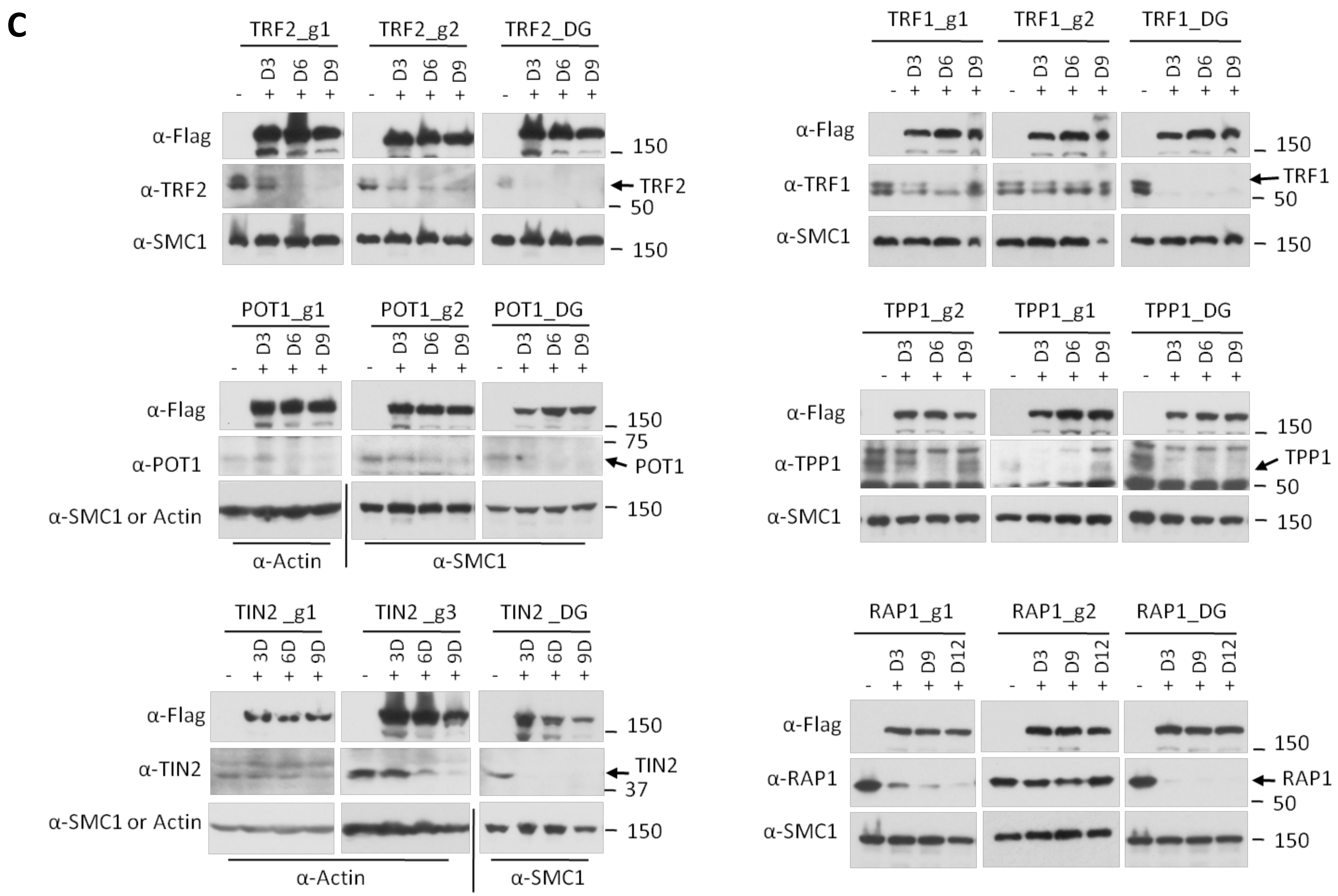
C
TRF2_g1
TRF2_g2
TRF2_DG
TRF1_g1
TRF1_g2
TRF1_DG
D3 D6 D9
α-Flag
α-TRF2
α-SMC1
TRF2
α-TRF1
TRF1
150
50
POT1_g1
POT1_g2
POT1_DG
α-POT1
POT1
α-SMC1 or Actin
α-Actin
α-SMC1
75
TPP1_g2
TPP1_g1
TPP1_DG
α-TPP1
TPP1
TIN2 _g1
TIN2 _g3
TIN2 _DG
3D 6D 9D
α-TIN2
TIN2
37
RAP1_g1
RAP1_g2
RAP1_DG
D3 D9 D12
α-RAP1
RAP1

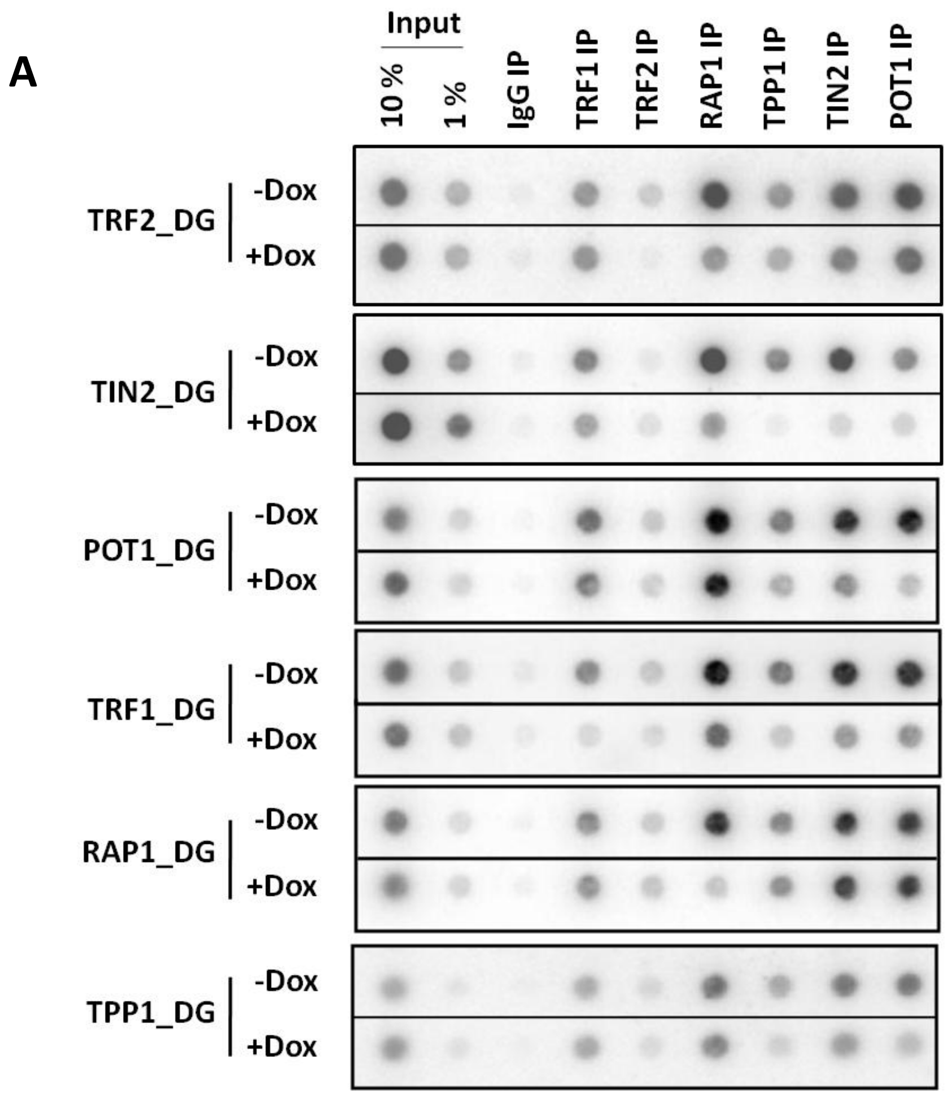
A
Input
10 %
1 %
IgG IP
TRF1 IP
TRF2 IP
RAP1 IP
TPP1 IP
TIN2 IP
POT1 IP
TRF2_DG
TIN2_DG
POT1_DG
TRF1_DG
RAP1_DG
TPP1_DG
-Dox
+Dox

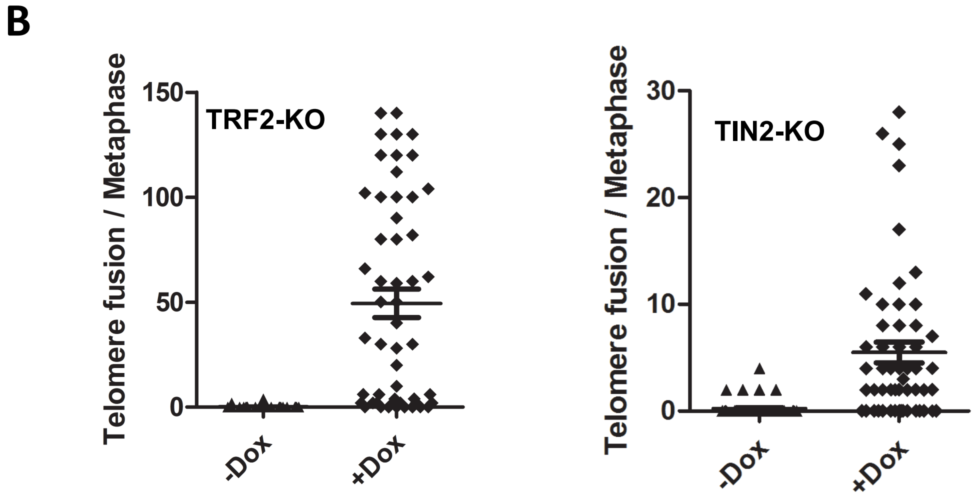
B
Telomere fusion / Metaphase
150
100
50
0
TRF2-KO
-Dox
+Dox
Telomere fusion / Metaphase
30
20
10
0
TIN2-KO
-Dox
+Dox

Kim et al, suppl. Figure S4

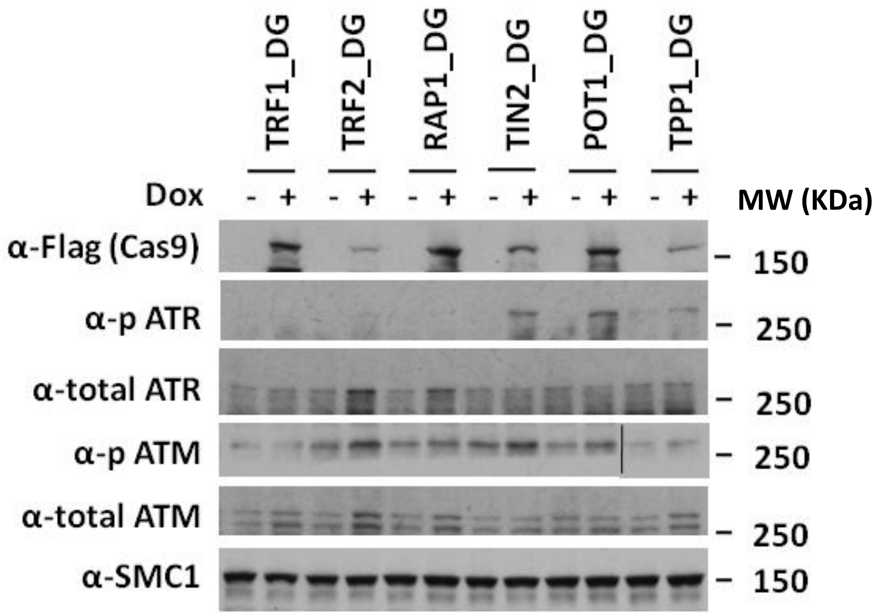

Kim et al, suppl. Figure S5

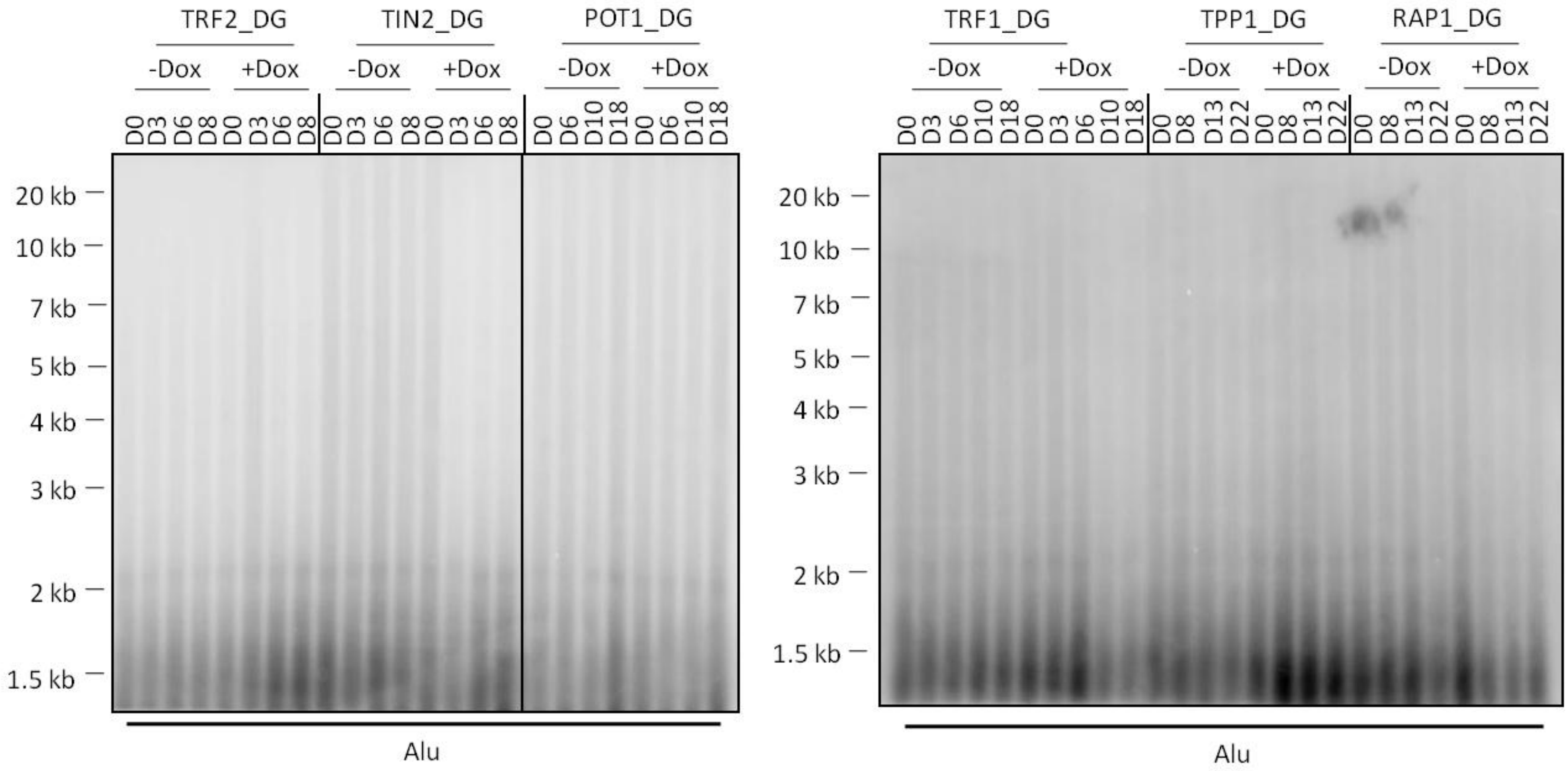

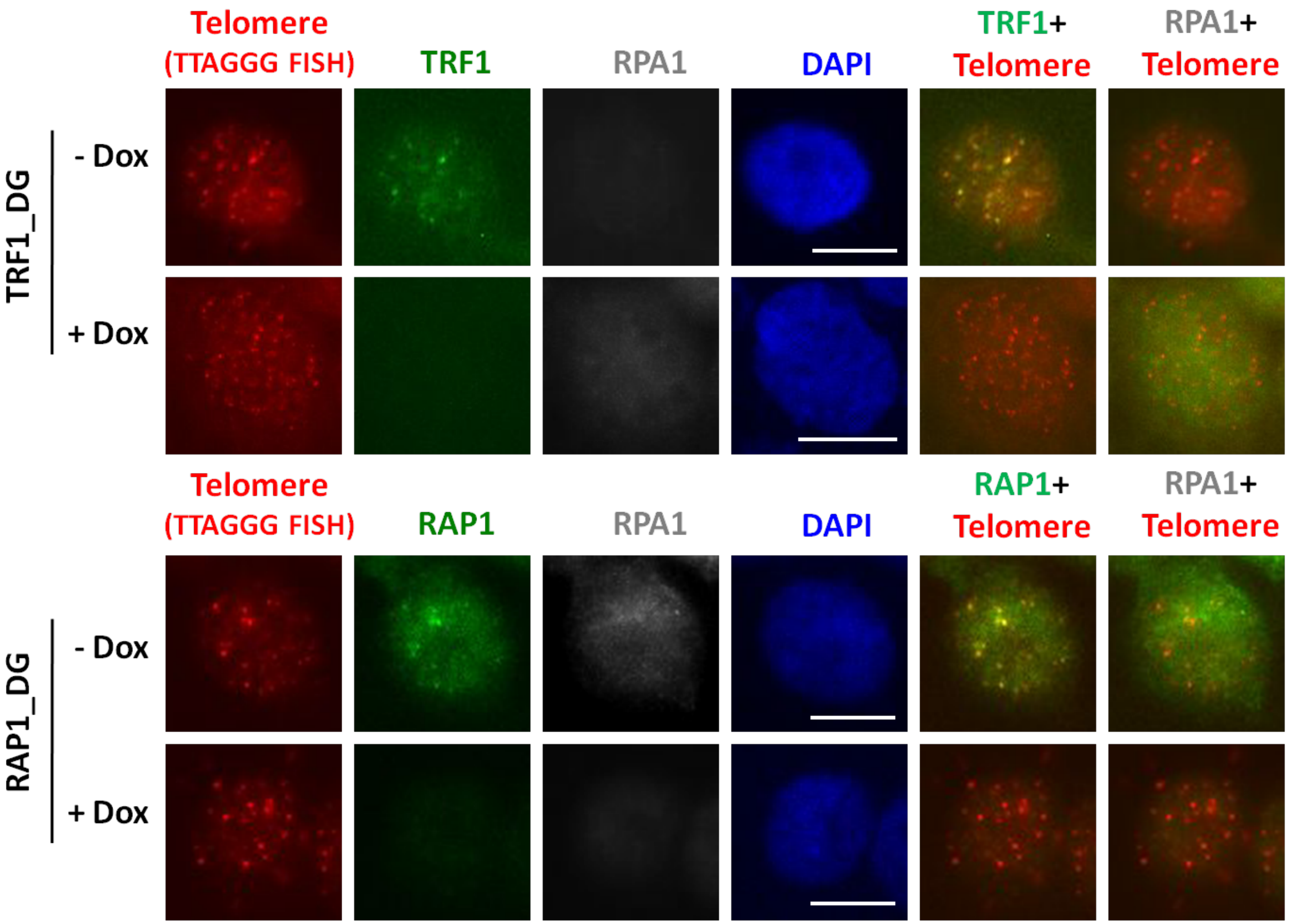
Telomere
(TTAGGG FISH)
TRF1
RPA1
DAPI
TRF1+
Telomere
RPA1+
Telomere
TRF1_DG
- Dox
+ Dox
Telomere
(TTAGGG FISH)
RAP1
RPA1
DAPI
RAP1+
Telomere
RPA1+
Telomere
RAP1_DG
- Dox
+ Dox

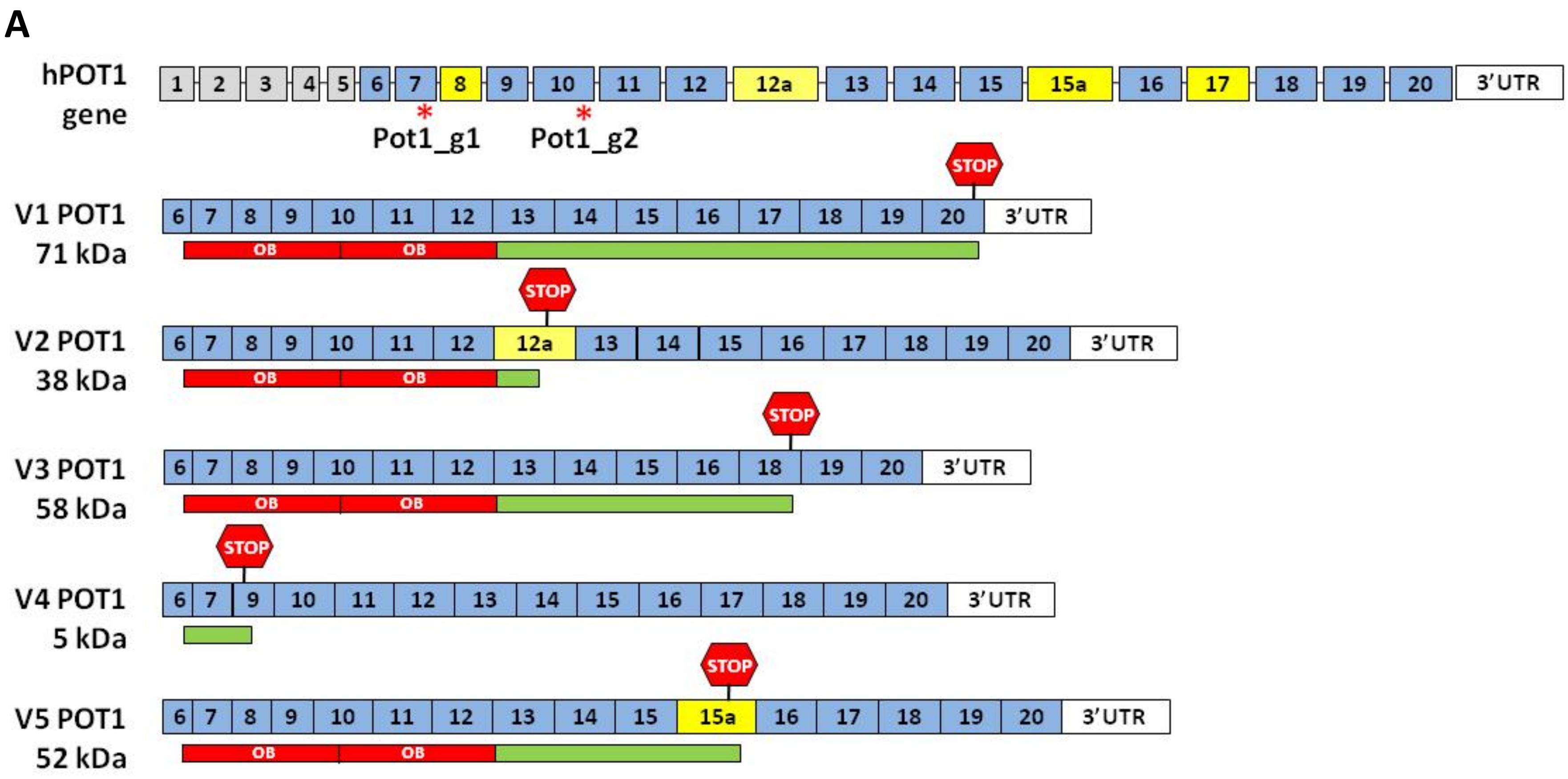
A
hPOT1 gene
1 2 3 4 5 6 7 8 9 10 11 12 12a 13 14 15 15a 16 17 18 19 20 3'UTR
Pot1_g1
Pot1_g2
V1 POT1 71 kDa
6 7 8 9 10 11 12 13 14 15 16 17 18 19 20 3'UTR
STOP
OB OB
V2 POT1 38 kDa
6 7 8 9 10 11 12 12a 13 14 15 16 17 18 19 20 3'UTR
STOP
OB OB
V3 POT1 58 kDa
6 7 8 9 10 11 12 13 14 15 16 18 19 20 3'UTR
STOP
OB OB
V4 POT1 5 kDa
6 7 9 10 11 12 13 14 15 16 17 18 19 20 3'UTR
STOP
V5 POT1 52 kDa
6 7 8 9 10 11 12 13 14 15 15a 16 17 18 19 20 3'UTR
STOP
OB OB

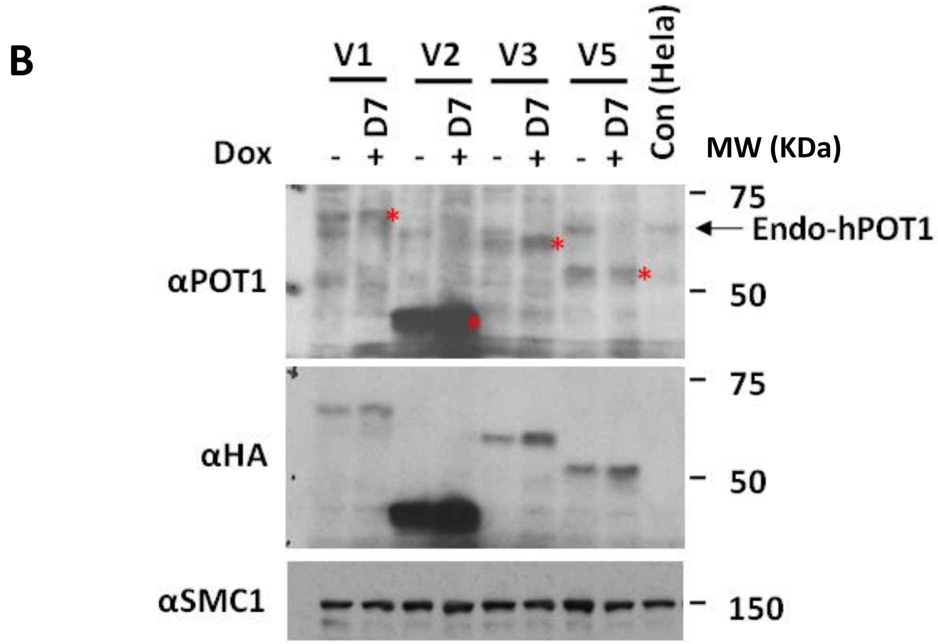
B
V1
V2
V3
V5
D7
D7
D7
D7
Con (Hela)
Dox
-
+
-
+
-
+
-
+
MW (KDa)
αPOT1
75
Endo-hPOT1
50
αHA
75
50
αSMC1
150

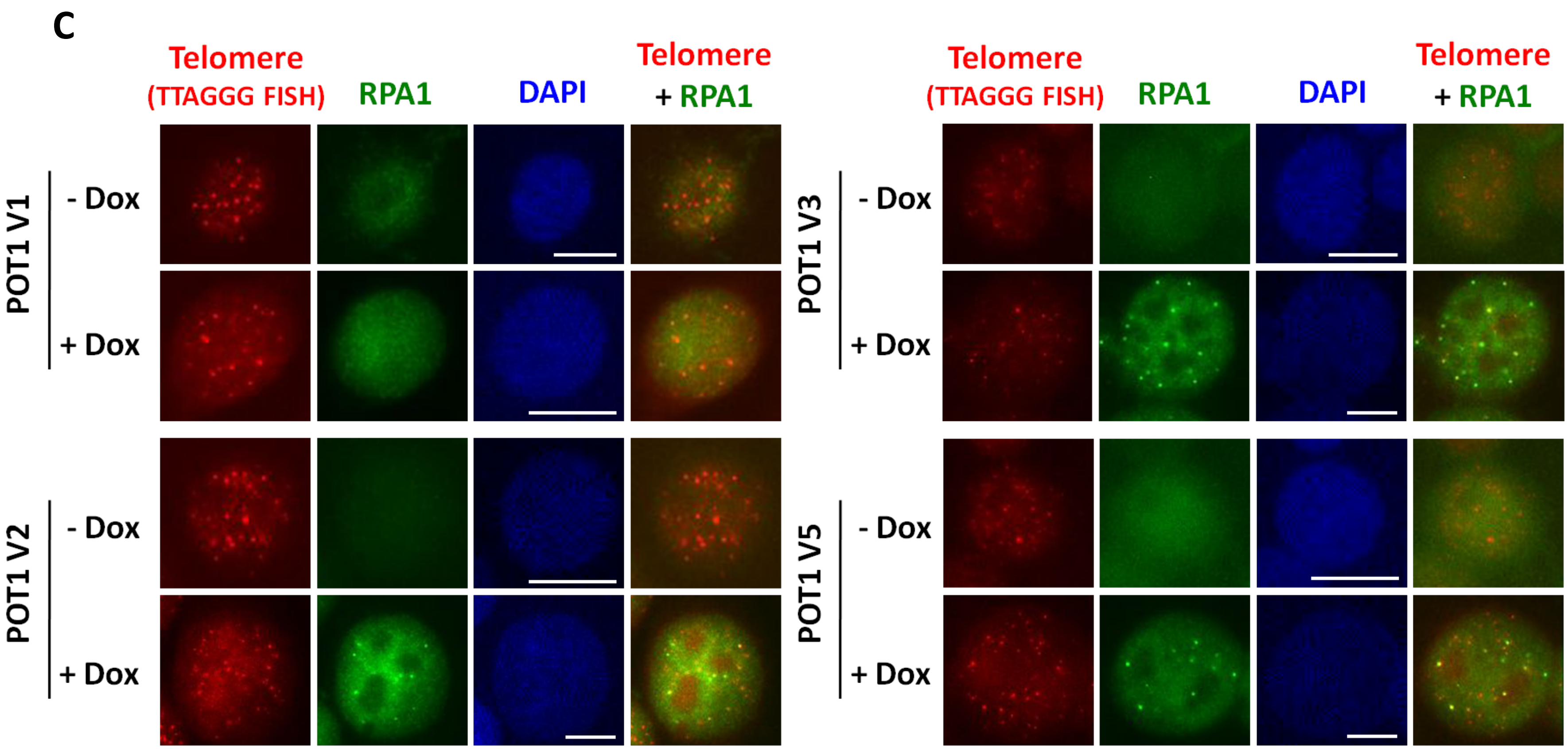
C
Telomere
(TTAGGG FISH)
RPA1
DAPI
Telomere
+ RPA1
POT1 V1
- Dox
+ Dox
POT1 V2
- Dox
+ Dox
POT1 V3
- Dox
+ Dox
POT1 V5
- Dox
+ Dox

D

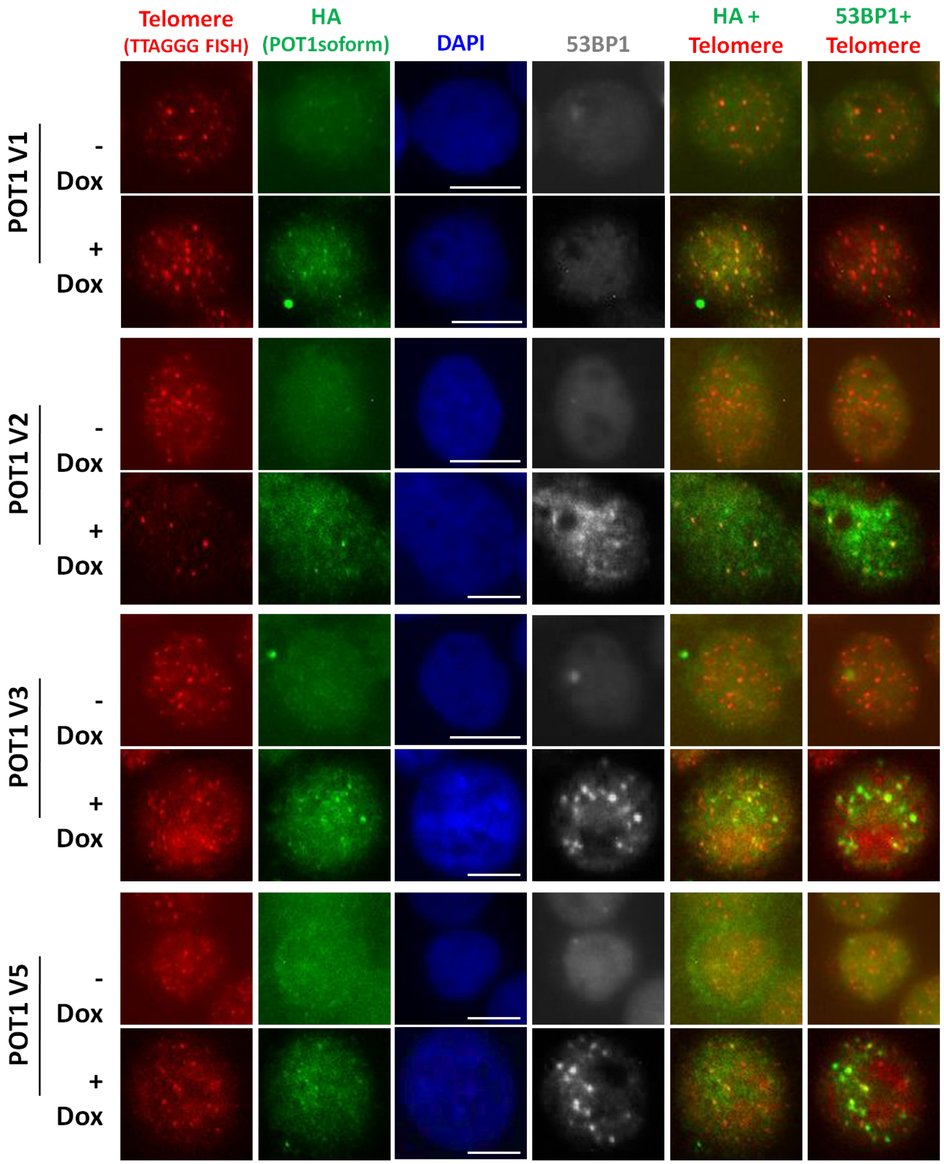
Telomere
(TTAGGG FISH)
HA
(POT1soform)
DAPI
53BP1
HA +
Telomere
53BP1+
Telomere
POT1 V1
POT1 V2
POT1 V3
POT1 V5
- Dox
+ Dox

Name of gRNA	Target sequence (5'----3')
TRF1 g1	TAGGGATGCCGACCCTACTG
TRF1 g2	AGAATTTGAGATCTGGCGTG
TRF2 g1	GCCTTTCGGGGTAGCCGGTA
TRF2 g2	CTGTGATGATTAAGGATCGC
RAP1 g1	CGGCGGCGGACACCGGCTCG
RAP1 g2	TAAGATCATTCGGCAGTTAA
TPP1 g1	GCCCCTGATACGTCCGACGT
TPP1 g2	TCTGTGCTCGGGTCCAAGCT
TIN2 g1	AGCTGCGGGACCCGCCACCA
TIN2 g2	GGCCCATACAAAGGCGTTCG
TIN2 g3	GAACTCTTCGTCGGCCTAGA
POT1 g1	GAGATATTGTTCGCTTTCAC
POT1 g2	GGAGGTACCAGTTACGGTCG

Name	sequence (5'----3')
TIN2 PCR forward primer	GGAGGAGCAACTTTGCTGCACC
TIN2 PCR reverse primer	ATGGAAGGGAGCCGAGTCAGATC

Name	Target sequence (5'----3')
TRF2 shRNA1	AGGAAATGGTGAAGTCTAT
TRF2 shRNA2	GAGCATGGTTCCTAATAAT
GFP shRNA	CACAAGCTGGAGTACAACT

Table S1. List of gRNAs, shRNAs, and primers used in the study.
